# Hepatoprotective action of *Sonchus oleraceus* against paracetamol-induced toxicity via Nrf2/KEAP-1/HO-1 pathway in relation to its metabolite fingerprint and *in silico* studies

**DOI:** 10.1371/journal.pone.0325782

**Published:** 2025-06-26

**Authors:** Mohamed F. Abdelhameed, Marawan A. El-Baset, Amira R. Khattab, Rehab F. Taher, Mohamed A. El-Saied, Asmaa S. Abd Elkarim, Ahmed F. Essa, Ahmed A. El-Rashedy, Mohamed A. Farag, Hiroshi Imagawa, Abdelsamed I. Elshamy, Ahmed M. Abd-ElGawad

**Affiliations:** 1 Pharmacology Department, Medical Research and Clinical Studies Institute, National Research Centre, 33 El Bohouth St., Dokki, Cairo, Egypt; 2 Stark Neurosciences Research Institute, Indiana University School of Medicine, Indianapolis, Indiana, United States of America; 3 Pharmacognosy Department, College of Pharmacy, Arab Academy for Science, Technology and Maritime Transport, Alexandria, Egypt; 4 Department of Natural Compounds Chemistry, National Research Center, 33 El Bohouth St., Dokki, Giza, Egypt; 5 Department of Pathology, Faculty of Veterinary Medicine, Cairo University, Giza, Egypt; 6 Chemistry of Tanning Materials and Leather Technology Department, National Research Centre, 33 El Bohouth St., Dokki, Giza, Egypt; 7 Chemistry of Natural and Microbial Products Department, National Research Centre, 33 El Bohouth St., Dokki, Giza, Egypt; 8 Department Organic and Medicinal Chemistry, Faculty of Pharmacy, University of Sadat City, Menoufia, Egypt; 9 Pharmacognosy Department, Faculty of Pharmacy, Cairo University, Cairo, Egypt; 10 Faculty of Healthcare, Saxony Egypt University (SEU), Badr city, Egypt; 11 Faculty of Pharmaceutical Sciences, Tokushima Bunri University, Yamashiro-cho, Tokushima, 770, Japan; 12 Plant Production Department, College of Food & Agriculture Sciences, King Saud University, P.O. Box 2460, Riyadh 11451, Saudi Arabia; Dr. Anjali Chatterji Regional Research Institute for Homeopathy, INDIA

## Abstract

**Background:**

Paracetamol overdose causes severe hepatotoxicity. *Sonchus oleraceus* is traditionally used to treat liver disorders, but its potential against paracetamol-induced liver injury is unexplored. This work aimed to investigate the protective mechanisms of an *S. oleraceus* extract (SOEtOH) using *in vivo*, histological and biochemical assessments along with metabolomics profiling and *in silico* studies, including molecular docking and dynamic simulations (MD).

**Methods and findings:**

SOEtOH was administered to rats with paracetamol-induced hepatotoxicity at 50, 100, and 200 mg/kg doses. Serum enzymes, hepatic antioxidants, and histopathology were evaluated. UPLC-MS characterized bioactive metabolites and molecular docking and assessed their anti-inflammatory potential. SOEtOH significantly restored serum ALT and AST toward normal levels in a dose-dependent manner. It also replenished depleted hepatic glutathione (up to 3.9-fold) and superoxide dismutase (up to 4.7-fold). Immunohistochemistry revealed SOEtOH progressively attenuated caspase-3 expression related to apoptosis. It also ameliorated characteristic histopathological alterations like necrosis, inflammation, and sinusoidal congestion. Thirty-two bioactive metabolites, including flavonoids, phenolic acids, and terpenes, were identified. Molecular docking revealed potent anti-inflammatory effects via JNK inhibition, with luteolin-*O*-dihexoside, isorhamnetin-*O*-hexoside, di-*O*-caffeoylquinic, and kaempferol-*O*-hexoside having the strongest binding affinities. MD simulations demonstrated that these compounds’ complexes significantly contribute to JNK1 and JNK2’s catalytic binding site.

**Conclusion:**

This integrated study demonstrates that SOEtOH protects against paracetamol hepatotoxicity by mitigating oxidative stress and inhibiting pro-inflammatory/apoptotic signaling. Our results reveal therapeutic lead compounds that may be further explored for clinical applications.

## 1. Introduction

Paracetamol, developed in 1878, is used as a pain relief and antipyretic agent. Studies link it to liver damage, especially at high doses. Underweight individuals, alcohol abusers, fasting individuals, and those with infections are more at risk [1]

Paracetamol is metabolized by the liver into non-toxic byproducts, but high doses can cause reactive oxygen species damage, with CYP2E1 being the leading cause [2]. Mitochondrial malfunction is the main cause of harmful free radicals and oxidative damage [3]. Hepatic damage typically begins 24–72 hours post-ingestion, leading to symptoms like jaundice, confusion, and hepatic failure [4].

Upon paracetamol metabolism by the liver, it yields a toxic metabolite called NAPQI. NAPQI can bind to mitochondrial proteins, which can disrupt the electron transport chain, leading to mitochondrial dysfunction. Mitochondrial dysfunction can cause a number of problems, including liver damage, kidney damage, and even death [5]. Increased activity of mitochondrial complex I is an indicator of free radical production, correlating with the severity of liver injury [3,5]. The primary sources of oxidative and nitrosative stress in paracetamol overdose are peroxynitrite and mitochondrial superoxide, with peroxynitrite being highly reactive and contributing significantly to cellular damage [6]. The antioxidant defense system is essential for protecting against cellular damage caused by free radicals, with glutathione (GSH), superoxide dismutase (SOD), Nrf2, Keap1, and HO-1 as crucial molecules in that defensive system [7]. So, the enrichment of the antioxidant system could play a vital role in the management of paracetamol-induced hepatotoxicity.

The native people of North America frequently consume plants from the Asteraceae genus *Sonchus*. Young green leaves can be eaten raw, whereas older ones can also be eaten after a brief boiling period [8]. One of the most important members of this genus worldwide is *S. oleraceus* L., owing to its nutritional and therapeutic value [9]. The most common way to eat *S. oleraceus* is by inclusion with sauces and broths [10]. Regarding its health benefits, several biological actions have been reported, including antihemolytic [11], cytotoxicity, antimicrobial [12], antidiabetic [13], anti-inflammatory [14], anti-nociceptive [15], anti-aging [16], antioxidant and anti-cholinesterase activities [17], alongside with wound healing properties [18]. *S. oleraceus* phytochemical components, such as flavonoids, phenolics, and sesquiterpene lactones, were renowned for their antioxidant and anti-inflammatory effects, two important processes in liver protection [12,13,17].

*Sonchus oleraceus* was selected over other hepatoprotective agents such as *Silybum marianum* and *Andrographis paniculata* due to its dual role as a traditional food and medicinal plant. Its rich phytochemical profile—including flavonoids, phenolic acids, and antioxidants [12,13,17]—has demonstrated significant hepatoprotective, anti-inflammatory [14], and antioxidant [17] activities in various studies. Unlike *S. marianum* and *A. paniculata*, which are primarily used as herbal supplements, *S. oleraceus* is consumed as a leafy vegetable [9], offering greater accessibility, dietary integration, and safety for long-term use. Moreover, its underexplored potential provides a novel avenue for developing food-based liver-supportive therapies. Early ethnopharmacological reports indicate a possible involvement in liver health, but complete scientific confirmation is missing. By addressing this research gap, the study hopes to reveal additional insights into the hepatoprotective effectiveness of *S. oleraceus*, potentially leading to the development of new natural therapy techniques for liver illnesses.

On the molecular level, a serine/threonine protein kinase, c-Jun N-terminal kinase (JNK), is a member of the mitogen-activated protein kinase (MAPK) superfamily. In mammals, JNK has been detected in three different isoforms. JNK1 and JNK2 are present all over the body; JNK3 is mostly prevalent in the brain and testicles [19]. Inflammation, steatosis, fibrosis, and hepatic cell death are among the liver maladies for which studies over the past few decades have provided compelling evidence of the detrimental effects of persistent JNK activation on hepatocyte mortality and liver damage [20]. It is interesting to note that JNK activation has also been connected to acetaminophen overdoses [21]. JNK activation under oxidative stress conditions leads to its translocation to the mitochondria and initiates a second blow that amplifies the initial oxidative stress, causing mitochondrial permeability transition pore formation, complete loss of the mitochondrial membrane potential, and, ultimately, cell necrosis [22]. Consequently, JNK is an important therapeutic target in the treatment of hepatotoxicity [23].

Therefore, this study’s main goals were to investigate i) the antioxidant activities of *S. oleraceus* ethanol extract (SOEtOH) against paracetamol-induced liver toxicity in rats, ii) the action mechanism of SOEtOH for mitigation against oxidative stress via biochemical, histochemical and immunohistochemical analysis, iii) the SOEtOH metabolites profiling using UPLC-ESI–Qtof-MS technique to identify underlying active chemicals, and iv) the roles of the main identified bioactive compounds *via* molecular docking study and dynamic simulations on the JNK as a main target in the hepatotoxicity assay.

## 2. Materials and methods

### 2.1. Plant authentication and collection

**Fig 1:**
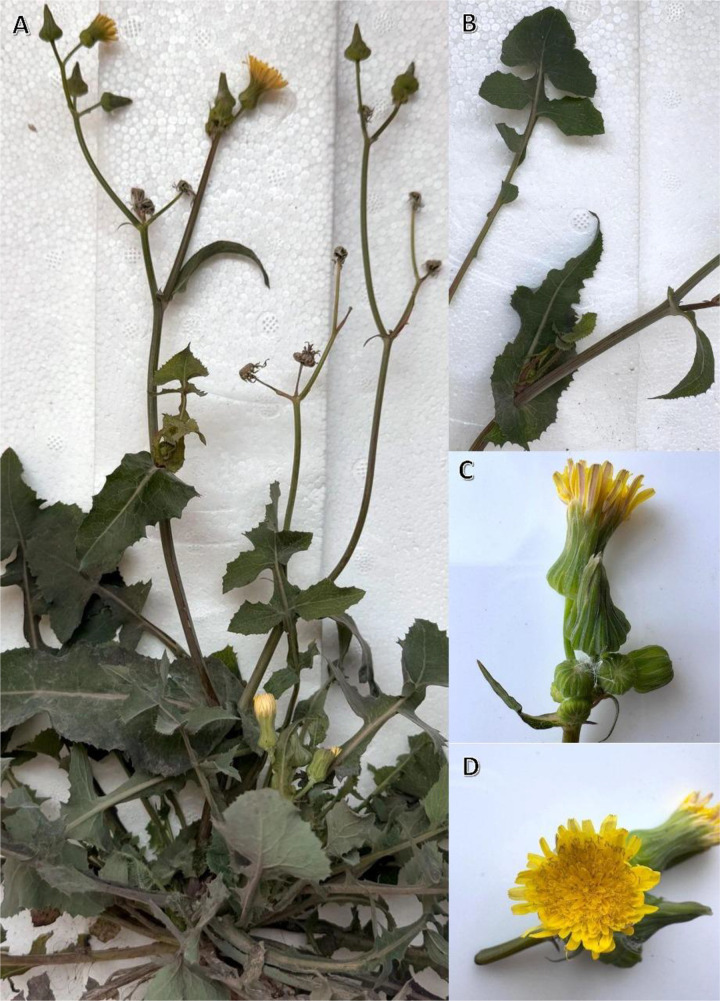
The herb *Sonchus oleraceus* L. A) Plant shoot system, B) close view of leaves, C and D) close view of closed and open flowers.

The *S. oleraceus* aerial parts (Fig 1) were gathered in early morning of 26 April 2022 during the blossoming season in the Menoufia region of the Nile Delta (30°21’01.0“N 30°52’11.9”E). The identification of the plants was verified by Prof. Dr. Ahmed Abdel Gawad, a taxonomy professor at Mansoura University. Mansoura University’s Herbarium held a voucher specimen (SO-xq-774z/21–02712).

### 2.2. Extraction procedure

The gathered *S. oleraceus* plant materials were placed in a dry, open, and shaded space to remove water at room temperature. Following thorough drying, the plant material was ground into powder using a plant grindery. The 70% ethanol was used to extract about 600 g, and the whole extract was gathered and dried with rotary evaporator (Heidolph, Germany) under reduced pressure at 50C to produce dark-black gum (SOEtOH: 26.8 g). This gum was then kept at 4 °C until additional chemical and biological tests were conducted. Ethanol 70% is a cost-effective, safe, and efficient solvent for extracting bioactive compounds due to its enhanced cell permeability, prolonged contact time, and balanced polarity.

### 2.3. Bioassays

#### 2.3.1. Drugs and chemicals.

Paracetamol was purchased from Julphar Pharmaceutical Co. (Dubai, UAE). Ethanol (96%) was purchased from Merck Millipore (Burlington, MS, USA). ALT, AST, and urea colorimetric kits were purchased from Bio Diagnostic (Giza, Egypt). GSH (# K464-100) and SOD (#K335-100) were measured using a kit from Bio Vision Inc. (San Francisco, CA, USA). Nuclear factor erythroid 2-related factor 2 (Nrf2, cat# SL0985Ra) was obtained from (Sunlong Biotech CO., Ltd.), Hangzhou, China and (HO-1, sc-136960), Santa Cruz Biotechnology, Inc., (Dallas, Texas, USA). KEAP-1 (KEAP-1, #EKL58562) ELISA kits were obtained from Biomatik (Wilmington, Delaware, USA).

#### 2.3.2. Experimental animals and ethical approval.

Twenty-five male Albino Wistar rats (180–200 g) were obtained from the National Research Center’s Animal Facility in Giza, Egypt. They were housed in pathogen-free cages under a controlled dark-light cycle and regulated room temperature. To acclimate, the rats were maintained under these conditions for two weeks before the experiment. All procedures adhered to the ethical guidelines for the Care and Use of Experimental Animals, as approved by the Institutional Animal Care and Use Ethical Committee (Vet. CU. IACUC) [Approval No: Vet CU 03162023658] along with fulfillment of ARRIVE’s guidelines.

#### 2.3.3. Experimental design and dosing regimen.

The acute toxicity of SOEtOH has been reported in multiple studies [15], all confirming its safety for mice and rats [24]. Accordingly, the rats were randomly assigned to five groups (n = 5) as follows: **Group 1 (Normal Control):** The rats received a 0.9% saline solution for 14 days. **Group 2 (Positive Control):** The rats received orally a single oral dose of paracetamol (640 mg/kg body weight) 24 hours before blood sampling [25]. **Groups 3, 4, and 5:** The rats were administered orally doses of 50, 100, or 200 mg/kg SOEtOH daily for 15 days followed by a single dose of paracetamol (640 mg/kg) on day 15. At the end of the study, sodium phenobarbital (140 mg/kg) was injected intraperitoneally to humanely end the lives of all the rats which were confirmed by cervical dislocation. Euthanasia was prompted by severe pain indicators such as abdominal guarding, stooped posture, reluctance to move, or excessive abdominal licking, especially if pain persisted despite analgesic treatment (meloxicam 2 mg/kg, i.p.). No premature deaths or unplanned fatalities occurred, and all animals remained in good health throughout. Proper disposal followed the National Research Center’s Safety and Health Committee guidelines.

#### 2.3.4. Blood collection and tissue preparation.

Blood was collected under light anesthesia from the retro-orbital plexus 24 h after paracetamol administration and transferred into sterile centrifuge tubes. After clotting, samples were centrifuged for 10 minutes at 1400 × g using a cooling centrifuge (Sigma & Laborzentrifugen Gmbh; Osterode am Harz, Germany; Model: 2 k15). The isolated Serum stored at −80 °C in Eppendorf tubes for ALT and AST analysis. Following anesthesia, livers were excised, weighed, and rinsed with cold saline. Tissue samples were homogenized in ice-cold PBS (10% w/v) and centrifuged at 1800 × g for 10 minutes at 4 °C. The resulting supernatants were used for biochemical assessments of GSH, SOD, Nrf2, HO-1, and KEAP-1 using ELISA kits per manufacturer instructions. The remaining liver samples were fixed in 10% neutral-buffered formalin for 72 hours for histological analysis.

#### 2.3.5. Histopathological examination of the liver tissue.

Liver tissue samples were fixed in 10% neutral-buffered formalin, processed through alcohol and xylene, and embedded in paraffin. Sections (5 µm thick) were prepared and stained with hematoxylin and eosin (H&E). Ten random microscopic fields per section were examined under a Leica DM4 B light microscope (Leica Microsystems, Switzerland) to evaluate hepatic lesion scores. Hepatocellular changes were assessed using the scoring system of Muhammad-Azam et al. [26], ranging from 0 (normal hepatocytes) to 5 (extensive hepatocyte loss). The percentage of affected hepatic lobules determined the grading as follows: Grade 0: Normal hepatic lobules; Grade 1: Mild changes (<25% affected); Grade 2: Mild to moderate (<50%); Grade 3: Moderate to severe (50–70%); Grade 4: Severe (>75%); and Grade 5: Extensive involvement of entire hepatic lobules.

#### 2.3.6. Immunohistochemistry assaying.

Immunohistochemistry was performed for the detection of active caspases-3 as a marker of apoptosis. Liver tissue sections were carried out on positively charged glass slides. Briefly, tissue sections were subjected to heat-induced epitope retrieval followed by peroxidase blockage and then incubated with a monoclonal antibody against cleaved caspase 3 as a primary antibody (diluted 1:100; ab32042, Abcam, USA). After washing, slides were incubated using the Dako EnVision™ FLEX detection system in accordance with the manufacturer’s instructions to detect the reaction. Positive expression was visualized as a brown color that was measured as area % using Olympus CellSens dimensions software (Olympus, Tokyo, Japan) **[27].**

### 2.4. UPLC-ESI–QTOF-MS/MS analysis

Ethanol-water (7:3, *v/v*) solvent (10 mL) was used to extract 1 g of air-dried powdered *S. oleraceus* using an ultrasonic bath for 1 h (Branson Ultrasonic Corporation, Danbury, CT, USA). Following filtration and centrifugation of the extract for 15 min, clear supernatant was aliquoted and used for UPLC-ESI-Qtof-MS analysis following the exact conditions described in El-Gendy et al. [28], El-Fadaly et al., [29]. The chromatographic separation was performed on an ACQUITY UPLC system (Waters, Milford, MA, USA) using an ACQUITY UPLC HSS T3 Column (100Å, 1.8 µm, 2.1 mm × 100 mm), with a full loop injection volume of 3.1 µL and a binary gradient elution at a flow rate of 150 µL/min. The gradient program was as follows: 0–1 min, isocratic 95% solvent A (water/formic acid, 99.9:0.1 v/v); 1–16 min, linear increase from 5% to 95% solvent B; 16–18 min, isocratic 95% B; 18–20 min, isocratic 5% B. Detection was conducted using an Agilent 6540 UHD Accurate-Mass Q-TOF LC/MS system with an ESI interface, acquiring data in both positive and negative ionization modes across an m/z range of 70–1100 at a scan rate of 2.5 Hz. The Mass Hunter Workstation software (version B.04.00) was used for data acquisition, with a 100-ppm detection window and compound identification based on accurate mass (≤10 ppm error), retention time, MS/MS fragmentation patterns, and comparison with reference standards when available. The method was validated and demonstrated excellent analytical performance, with intra-day and inter-day precision (expressed as %RSD) below 5% for all tested analytes, accuracy ranging from 95% to 105% across low, medium, and high concentration levels, and reproducibility consistently within 5% RSD, confirming the robustness and reliability of the UPLC-MS method for high-confidence qualitative and quantitative analysis.

### 2.5. *In silico* studies

#### 2.5.1. Molecular docking studies.

The docking process of the main phenolics and flavonoids, including hydroxy-tetramethoxyflavone (2), protocatechuic acid hexoside (3), caffeic acid-*O*-hexoside (4), dihydroxycoumarin-*O*-hexoside (5), caffeic acid (6), dihydroxycoumarin (7), luteolin-*O*-di-hexoside (8), kaempferol-*O*-hexoside (9), luteolin-*O*-hexoside (10), 3,4-di-*O*-caffeoylquinic acid (11), isorhamnetin-*O*-hexoside (12), apigenin-*O*-glucoronide (13), luteolin (19), kaempferol (21), caffeic acid ethyl ester (22), and apigenin (24), identified using UPLC-MS was carried out using the version 2015.10 of Molecular Operating Environment (MOE) software. The catalytic domains of c-Jun NH_2_-terminal kinases (JNK1 and JNK2) (PDB ID: 3V3V and 3NPC), respectively [30], were obtained from the Protein Data Bank (PDB). Further, extra solvent and cofactors were removed from the proteins using the default MOE “QuickPrep” module parameters. Additionally, the ‘Site Finder’ feature was used to find the potential binding pockets, including the crucial amino acid residues. The docking process has been validated by re-docking of the ligands in their binding site. The prepared compounds were docked in the binding site, and the results were presented as ΔG ^*a*^ (kcal/mol) with RMSD values of 2. Also, the 2D and 3D representations of the best conformers were visualized using *BIOVIA* discovery studio visualizer 21.1.0.0.

#### 2.5.2. Molecular dynamic (MD) simulations.

Through incorporating molecular dynamic (MD) simulations into the examination of biological systems, it is possible to investigate the mechanical movement of atoms and molecules that is not readily accessible via other approaches [31]. The knowledge gained from running this simulation offers a complex viewpoint on the dynamical development of biological systems, including molecular interaction and modification of conformation [31]. The AMBER 18 package’s GPU version of the PMEMD engine was used to run the MD simulations for every system [32].

The General Amber Force Field (GAFF) method from ANTECHAMBER was used to determine each compound’s partial atomic charge [33]. Within 10 Å of every box edge, the Leap component of the AMBER 18 package automatically dissolved every system inside an orthorhombic box of TIP3P water molecules. Each system was neutralized by adding Na^+^ and Cl^-^ counter ions using the Leap module. Each system underwent a 2000-step initial reduction with a 500 kcal/mol imposed restraint prospective, and then a 1000-step complete reduction utilizing the conjugate gradient algorithm without constraints.

To guarantee that every system had the same number of atoms and volume, the MD simulation involved progressively heating each system from 0K to 300K over 500 ps. The solutes in the system were subjected to a collision frequency of 1 ps and a potential harmonic constraint of 10 kcal/mol. Each system was then heated to a uniform temperature of 300K and allowed to equilibrate for 500 ps.The number of atoms and pressure in each system were kept constant for each production simulation in order to model an isobar-ic-isothermal (NPT) ensemble. The Berendsen barostat was used to keep the system’s pressure at 1 bar [34].

Every system underwent MD simulation for 40 ns. In each simulation, hydrogen’s bond atoms were constrained using the SHAKE approach. A 2 fs step size and an SPFP accuracy model were incorporated into each simulation. The simulations were conducted using an isobaric-isothermal ensemble (NPT) with randomly seeding, a Langevin thermostat with a collision frequency of 1 ps, a temperature of 300K, a constant pressure of 1 bar, and a pressure-coupling constant of 2 ps.

#### 2.5.3. Post-MD analysis.

The CPPTRAJ module of the AMBER18 suite [35] was used to investigate the trajectories that were acquired by MD simulations after they were saved every 1 ps. The entire graphs and visualizations were made using Chimera [36] and the Origin data analysis tool [37].

#### 2.5.4. Thermodynamic calculation.

In order to estimate ligand-binding affinities, the Poisson-Boltzmann or generalized Born and surface area continuum solvation (MM/PBSA and MM/GBSA) technique has proven to be helpful. Within a specified force field, the Protein-Ligand complex molecular simulations utilized by MM/GBSA and MM/PBSA calculate exact statistical-mechanical binding free energy [38]. Averaging the binding free energy across 400 images taken from the full 40 ns trajectory. For any molecular species (complex, ligand, and receptor), an estimate of the variation in binding free energy (ΔG) might be shown as follows [39].


$$\Delta G_{bind}=G_{complex}-G_{receptor}-G_{ligand}$$ (1)

$$\Delta G_{bind}=E_{gas}+G_{sol}-TS$$ (2)

$$E_{gas}=E_{int}+E_{vdw}+E_{ele}$$ (3)

$$G_{sol}=G_{GB}+G_{SA}$$ (4)

$$G_{SA}=\gamma SASA\;$$ (5)

The gas-phase, internal, Coulomb, and van der Waals energies are denoted by the letters Egas, Eint, Eele, and Evdw. The FF14SB force field terms were used to directly evaluate the Egas. The energy involved in both polar states (GGB) and non-polar states (G) was used to calculate the solution-free energy (Gsol). Using a water probe radius of 1.4 Å, the non-polar solvation free energy (GSA) was calculated using the Solvent Accessible Surface Area (SASA) [40]. On the other hand, the polar solvation (GGB) contributions were evaluated by solving the GB equation. Items S and T represent the solute’s total entropy and temperature, respectively. Each residue’s contribution to the overall binding free energy was determined using Amber18’s MM/GBSA-binding free energy approach.

### 2.6. Statistical analysis

The results in the current study were presented as mean ± SEM. One-way analysis of variance (ANOVA) was used for the data analysis, and Tukey’s multiple comparison test was used to assess the significance of the average variation between the groups. Using the software program GraphPad Prism (version 9.00; GraphPad Software, Inc., San Diego, CA, USA), a *p*-value of 0.05 or less was deemed statistically significant.

## 3. Results

### 3.1. Biological evaluations results

#### 3.1.1. Effect of SOEtOH on paracetamol-associated changes in ALT and AST levels.

**Fig 2:**
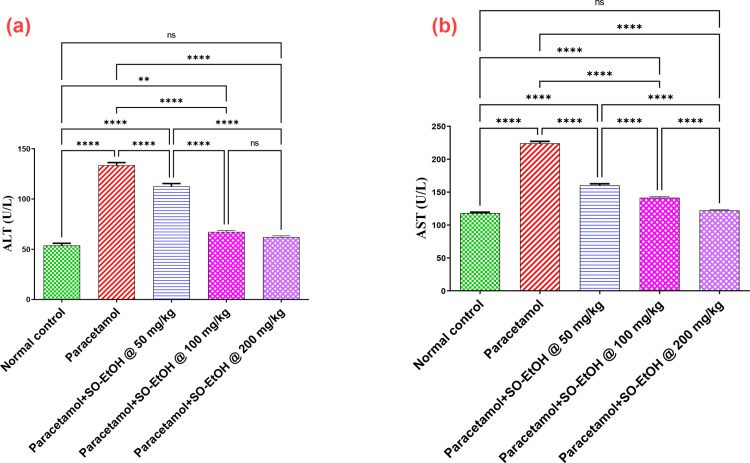
Effect of SOEtOH on serum ALT and AST levels of paracetamol intoxicated rats. ALT (A), AST (B). Each bar represents the mean ± SEM (n = 5). Statistical analysis was carried out by one-way analysis of variance (ANOVA) and followed by Tukey’s multiple comparison test.* *p* ≤ 0.05, ** *p* ≤ 0.01, *** *p* ≤ 0.001, **** *p* ≤ 0.0001, ns: non-significant, (SOEtOH) *Sonchus oleraceus* extract.

The intoxication of paracetamol led to a elevated levelsin ALT and AST serum levels by 148.3 and 90.1% compared with normal rat control. In contrast, rats treated with SOEtOH50, 100 or 200 mg/kg attained much lower ALT and AST levels at 84.3, 50.4, 46.5% and 71.3, 63, 54.3% in relation to paracetamol intoxicated group, respectively (Fig 2A & B). Noteworthy, SOEtOH100 or 200 mg/kg significantly retracted the elevation of liver enzymes (ALT and AST) compared to SOEtOH-50.

#### 3.1.2. Effect of SOEtOH on paracetamol-associated changes in GSH, SOD, Nrf2, Keap1, and HO-1 levels.

**Fig 3:**
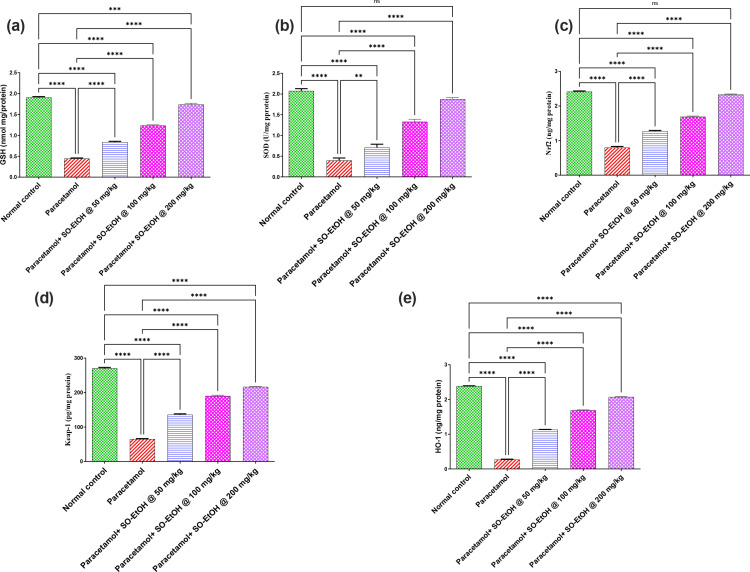
Effect of *S. oleraceus* extract (SOEtOH) on hepatic content of paracetamol intoxicated rats. (**A**) GSH, (**B**) SOD, (**C**) Nrf2, (**D**) Keap1 and (**E**) HO-1. Each bar represents the mean ± SEM (n = 5). Statistical analysis was carried out by one-way analysis of variance (ANOVA) and followed by Tukey’s multiple comparison test. * *p* ≤ 0.05, ** *p* ≤ 0.01, *** *p* ≤ 0.001, **** *p* ≤ 0.0001, ns: non-significant, (SOEtOH) *Sonchus oleraceus* extract.

Both hepatic levels of GSH and SOD activity of paracetamol-intoxicated rats were reduced by 77 and 81% compared to normal rats. In contrast, administration of SOEtOH-50, 100, or 200 at these dose levels replenished GSH hepatic level by1.9, 2.8, and 3.9-fold, and SOD activity by 1.8, 3.4, and 4.7-fold compared with paracetamol intoxicated rats (Fig 3A & B). Meanwhile, the protein content of Nrf2, Keap1, and HO-1 was reduced by 66, 76, and 89% compared with paracetamol-intoxicated rats. In contrast, SOEtOH-50, 100, or 200 ameliorated reduction in liver content of Nrf2 by 1.6, 2.1, and 2.9-fold, Keap1 by 2.1, 2.91 and 3.3-fold, and HO-1 by 4.2, 6.3 and 7.7-fold in relation with paracetamol intoxicated rats (Fig 3C–3E).

#### 3.1.3. Effects of SOEtOH on paracetamol‑induced histopathological alterations in hepatic tissues.

**Fig 4:**
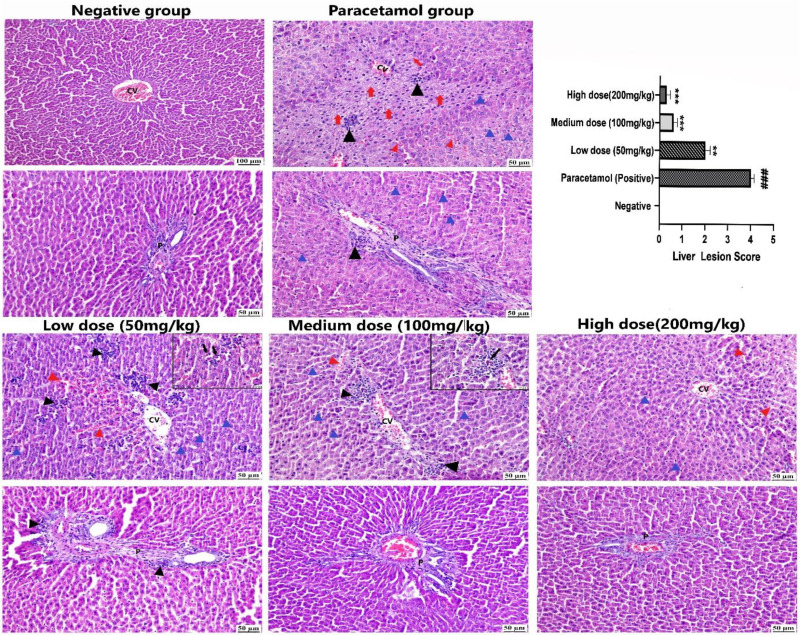
Histopathological examination of liver from different experimental groups (H&E).

As demonstrated in Fig 4, normal histology of hepatocytes and portal triad was found in the liver tissue of the control negative group. In contrast, as expected, histopathological examination of the paracetamol-treated group showed severe centrilobular vacuolar degeneration and hepatocellular necrosis that occupied almost all hepatic lobules, with various nuclear changes as karyopyknosis and karyolysis in addition to congestion of central veins and hepatic sinusoids. Marked activation of Kupffer cells with hyperplasia was prominent. Moreover, mononuclear inflammatory cells were infiltrated either between necrosed hepatocytes or within the portal area. In the low dose (50 mg/kg) group of SOEtOH hepatic sections exhibited vacuolar degeneration, sinusoidal congestion, multifocal aggregation of mononuclear inflammatory cells with hyperplasia of Kupffer cells, obvious hepatocellular apoptosis, and mild periportal leukocytic infiltration was detected. Likewise, in the medium dose group (100 mg/kg), mild degenerative changes of hepatocytes, sinusoidal congestion with mononuclear aggregates, and scattered apoptotic bodies were observed, as well the portal triad was apparently normal. Marked improvement was detected in the high dose group (200 mg/kg) as manifested by apparently normal portal triad structure and hepatocytes in the examined hepatic lobules, except minimal vacuolar degeneration and sinusoidal congestion in some instances. The hepatic injury was subsided with medium and high doses of SOEtOH that were in accordance with histopathological alternations. A higher lesion score was observed in the paracetamol-treated group compared to other experimental groups; there was a significant difference between the low-dose group and both medium and high-dose groups; moreover, no statistically significant difference was detected between medium and high-dose groups.

Normal hepatocytes arranged in hepatic cords around normal central vein (cv) of control negative group, normal portal triad (p) of control negative group, paracetamol treated group showing extensive hepatocellular degeneration and necrosis (red arrow), multifocal aggregation of mononuclear inflammatory cells (black head arrow), hyperplasia of Kupffer cells (blue head arrow) and congestion of central vein and hepatic sinusoids (red head arrow) also portal triad (p) display mononuclear inflammatory cells infiltration (black head arrow), low dose group (50 mg/kg) exhibit sinusoidal congestion (red head arrow) with multifocal mononuclear cells aggregates in addition to Kupffer cells hyperplasia and hepatocellular apoptosis (insert; note the apoptotic bodies (black arrow)), with scanty mononuclear cells (black head arrow) at the portal area, Medium dose group (100 mg/kg) showing mild sinusoidal congestion (red head arrow), pericentral mononuclear cell infiltration (black head arrow) with scattered apoptotic cells (Insert; note the apoptotic bodies (black arrow)) in addition to apparently normal portal area of medium dose group. The high dose group (200 mg/kg) represents apparently normal portal area and hepatocytes with sinusoidal congestion (redhead arrow) and minimal vacuolation of some hepatocytes. (Scale bar: 50 µm). The chart presents the hepatic lesion score; the values are expressed as the mean ± SEM (n = 5) using one-way factorial analysis of variance (ANOVA), **###** *p* < 0.01 related to the negative group and ** *p* < 0.01, *** *p* < 0.001 related to paracetamol treated group.

#### 3.1.4. Effects of SOEtOH on caspase-3 expression in paracetamol‑induced liver injury.

**Fig 5:**
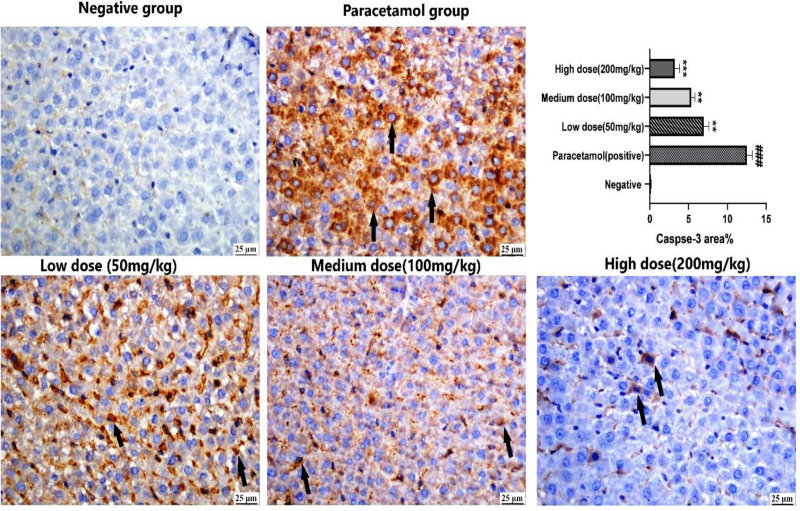
Immunoreactivity of active caspase-3 protein in hepatocytes of experimental groups.

Negative immunoreactivity against cleaved caspase-3 was detected in the control negative group. In contrast to the paracetamol-treated group, which showed strong positive reactivity against cleaved caspase-3 in the cytoplasm of hepatocytes, immunoreactivity was found to moderate in the low dose group that gradually decreased in medium and high dose groups, respectively. Evaluation of caspase 3 area % revealed a significant difference between experimental groups, with decreased expression in all treated groups compared with the paracetamol group. Treatment with SOEtOH reduced caspase-3 expression in a dose-dependent manner. Interestingly, there was no significant difference between the low and medium-dose groups. The high-dose group possessed significantly lower caspase-3 expression (Fig 5).

Control negative group showing no immune reactivity in hepatocytes, paracetamol group showing strong expression of caspase-3 in the cytoplasm of hepatocytes (arrows), low dose group (50 mg/kg) showing moderate immune expression (arrows), The expression gradually decreased in medium and high dose groups respectively (arrows), chart present caspase-3 area %, values are expressed as the mean ± SEM (n = 5) using one-way factorial analysis of variance (ANOVA), **###** *p* < 0.01 related to the negative group and ** *p *< 0.01, *** *p *< 0.001 related to paracetamol treated group.

### 3.2. Metabolites profiling of SOETOH extract via UPLC-PDA-ESI–qTOF-MS/MS

**Fig 6:**
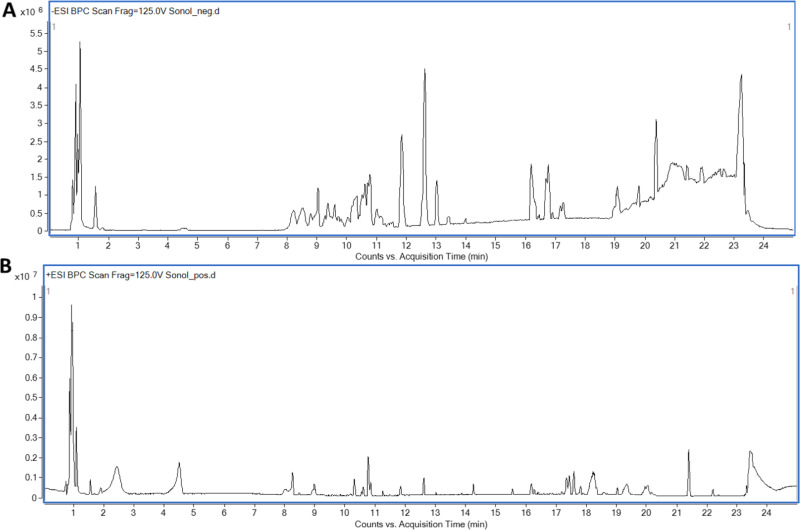
UPLC-qTOF-MS/MS base peak chromatograms of *S. oleraceus* ethanolic extract. Negative (A) and positive (B) ionization modes.

Ultra-high-performance liquid chromatography (UHPLC) coupled to mass spectrometry operated in +ve and -ve ion modes were performed for metabolites profiling of. SOEtOH extract to identify chemical compounds likely to mediate for the underlying hepatoprotective action (Fig 6). Results led to the assignment of 32 metabolites. Negative-ionization mode allowed for the detection of polar phenolic acids, glycosides, and fatty acids at higher sensitivity and lower signal-to-noise ratio, Metabolites belonged to various classes,*viz.* phenolic acids, flavonoids, coumarins, fatty acids,and terpenes (Table 1).

**Table 1:** Identified metabolites in SOEtOH via UPLC-qTOF-MS/MS in negative and positive ionization modes.

No	Rt.	Identification	Class	Mol. Ion *m/z* (±)	M.F	Error ppm	MS/MS	Ref
1	0.975	Unknown lignan	Lignan	377.0861^-^	C_18_H_17_O_9_	9.76	341, 317, 281, 215, 179, 161, 119, 89	[41]
2	7.415	Hydroxy-tetramethoxyflavone	Flavonoid	357.0869^-^	C_19_H_17_O_7_	5.04	299, 223, 151, 56	[42]
3	7.498	Protocatechuic acid-*O*-hexoside	Phenolic acid	315.0744^-^	C_13_H_15_O_9_	−4.12	274, 252, 153, 112	[43]
4	8.092	Caffeic acid-*O*-hexoside	Phenolic acid	341.0895^-^	C_15_H_17_O_9_	−6.69	295, 221, 179, 161, 135, 59	[44]
5	8.092	Dihydroxycoumarin-*O*-hexoside	Coumarin	341.0901^+^	C_15_H_17_O_9_	−6.69	295, 221, 179, 161, 135, 59	[45]
6	9.095	Caffeic acid	Phenolic acid	179.0352^-^	C_9_H_7_O_4_	−2.77	155, 135, 79	[46]
7	9.095	Dihydroxycoumarin	Coumarin	179.0355^+^	C_9_H_7_O_4_	−2.77	155, 135, 79	[45]
8	9.603	Luteolin-*O*-di-hexoside	Flavonoid	609.1463^-^	C_27_H_29_O_16_	0.47	542, 489, 447, 361, 306, 285, 227, 183, 143, 89	[47]
9	10.154	Kaempferol-*O*-hexoside	Flavonoid	447.0934^-^	C_21_H_19_O_11_	−0.43	401, 327, 285, 256, 179, 61	[47]
10	10.154	Luteolin-*O*-hexoside	Flavonoid	447.0935^+^	C_21_H_19_O_11_	−0.43	401, 327, 285, 256, 179, 61	[48]
11	10.419	Di-*O*-caffeoylquinic acid	Phenolic acid	515.1198^-^	C_25_H_23_O_12_	−0.65	449, 394, 353, 285, 191, 135, 59	[49]
12	10.593	Isorhamnetin-*O*-hexoside	Flavonoid	477.104^-^	C_22_H_21_O_12_	−0.1	413, 357, 315, 243, 163, 89	[50]
13	10.749	Apigenin-*O*-glucoronide	Flavonoid	447.0906^+^	C_21_H_19_O_11_	3.51	345, 271, 243, 207, 153, 107, 73	[46]
14	10.802	Dihydroxyoctadecenoic acid	Hydroxy fatty acid	311.1172^-^	C_18_H_33_O_4_	−8.61	268, 209, 148, 96	[48]
15	10.923	Unknown	Unknown	313.0534^+^	C_13_H_13_O_9_	9.94	272, 211, 152, 112, 57.0349	
16	11.270	Unknown	Unknown	193.0509^-^	C_10_H_9_O_4_	−2.17	161, 134, 106, 56	
17	11.404	Melampolide(costunolide)	Terpene	233.1500^+^	C_15_H_21_O_2_	15.5	187, 178, 159, 131, 107, 76	[51]
18	11.548	Trihydroxyoctadecadienoic acid	Hydroxy fatty acid	327.2180^-^	C_18_H_31_O_5_	−2.4	309, 283, 265, 171, 79	[52]
19	11.752	Luteolin	Flavonoid	285.0407^-^	C_15_H_9_O_6_	−0.77	241, 199, 133, 65	[46]
20	11.756	Hydroxynonadecadienoic acid	Hydroxy fatty acid	311.2626^+^	C_19_H_35_O_3_	−11.02	273, 224, 203, 174, 135, 113, 72	[53]
21	11.814	Kaempferol	Flavonoid	287.0548^+^	C_15_H_11_O_6_	1.06	269, 153, 115, 67	[47]
22	12.203	Caffeic acid ethyl ester	Phenolic acid	207.0665^-^	C_11_H_11_O_4_	0.81	179, 161, 135, 96	[54]
23	12.428	Unknown	Unknown	607.3006^+^	C_28_H_47_O_14_	−5.94	501, 435, 343, 257	
24	12.482	Apigenin	Flavonoid	269.0458^-^	C_15_H_9_O_5_	−0.81	225, 183, 117, 65	[48]
25	13.146	Hydroxy-oxo-phytodienoic acid	Hydroxy fatty acid	307.1917^-^	C_18_H_27_O_4_	0.18	289, 223, 185, 137, 109, 71	[55]
26	13.285	Dihydroxyoctadecanoic acid	Hydroxy fatty acid	287.2229^-^	C_16_H_31_O_4_	−1.33	223, 187, 149, 112, 63	[53]
27	15.563	Epoxy-hydroxy-octadecadienoic acid	Hydroxy fatty acid	311.2186^+^	C_18_H_31_O_4_	11.54	294, 268, 254, 213, 205, 184, 141, 118	[48]
28	16.112	Octadecatetraenoic acid	Fatty acid	277.2158^+^	C_18_H_29_O_2_	5.2	235, 171, 121, 93	[48]
29	16.227	Unknown nitrogenous lipid	Nitrogenous lipid	284.2909^+^	C_18_H_38_NO	13.78	204, 173, 97	
30	16.845	Hydroxyoctadecadienoic acid	Hydroxy fatty acid	295.2283^-^	C_18_H_31_O_3_	−0.99	277, 251, 195, 171, 113, 59	[48]
31	18.708	Hydroxynonadecatrienoic acid	Hydroxy fatty acid	309.2442^+^	C_19_H_33_O_3_	−5.6	221, 188, 156, 139, 111, 87	[53]
32	18.724	Maslinic acid	Terpene	473.3588^+^	C_30_H_49_O_4_	8.42	455, 407, 184	[56]

#### 3.2.1. Phenolic acids.

MS/MS spectral data obtained in negative ionization mode led to the assignment of several phenolic acids*viz*. protocatechuic acid hexoside (peak 3; *m/z* 315.0744, C_13_H_15_O_9_^-^),caffeic acid-*O*-hexoside (4) (peak 4; *m/z* 341.0895, C_15_H_17_O_9_^-^), to yield daughter ions at *m/z* 153 and 179, respectively due to their loss of hexose moiety.Peaks 11 was assigned as 3,4-*di-*caffeoylquinic acid (*m/z* 515.1198, C_25_H_23_O_12_^-^) based on fragment ion at *m/z* 353.088 [M-H-caffeoyl]^-^ and the diagnostic fragment at *m/z* 135 as observed in disubstituted quinic acid skeleton. Peaks 6 and 22 were assigned as caffeic acid (*m/z* 179.0352, C_9_H_7_O_4_^-^) and its ethyl ester (*m/z* 207.0665, C_11_H_11_O_4_^-^) [54].

Protocatechuic acid is reported to exhibit hypoglycemic, insulin-sensitizing, hypolipidemic, and antioxidant effects and mitigate dexamethasone-induced liver steatosis with the associated reduction in serum ALT and AST [57], and to likely contribute to the hepatoprotective effect against paracetamol toxic effect. Likewise, caffeic acid possesses antioxidant and hepatoprotective action as well as potential action in reducing DNA damage, thus reducing oxidative stress mediated by ethanol in rats [58].

#### 3.2.2. Coumarins.

One coumarin assigned as dihydroxycoumarin (peak 7; *m/z* 179.0355, C_9_H_7_O_4_^+^) was detected in positive MS ionization mode from fragment ion at *m/z* 103, corresponding to [M + H-44]^+^ loss of CO_2_ alongside with its *O*-hexoside derivative (peak 5; *m/z* 341.0901, C_15_H_17_O_9_^+^),with a loss of hexose to yield fragment ion at *m/z* 179 [66]. Dihydroxycoumarin, such as esculetin (6,7-dihydroxycoumarin), is well recognized for its hepatoprotective effect against CCl_4_-induced hepatic damage *via* reducing the level of hepatic injury biomarkers *viz.*gamma-glutamyl transpeptidase (GGT), lactate dehydrogenase (LDH) levels in serum [59].

#### 3.2.3. Flavonoids.

Flavonoids amounted to the major secondary metabolites class in SOEtOH. Five *O*-type flavonoid glycosides were identified *viz.* luteolin-*O*-di-hexoside (peak 8, *m/z* 609.1463, C_27_H_29_O_16_^-^) with a pseudomolecular ion at *m/z* 447 showing typical loss of a hexose moiety (162 Da), leading to an aglycone ion at *m/z* 285, which is also produced as a daughter ion from the fragmentation of luteolin-*O*-hexoside (peak 9, *m/z* 447.0934, C_21_H_19_O_11_^-^). Isorhamnetin-*O*-hexoside(peak 12, *m/z* 477.104, C_22_H_21_O_12_^-^) yielded a fragment ion of isorhamnetin at *m/z* 315 due to the loss of hexose moiety. Peak 13, with a molecular ion peak at *m/z* 447.0906 (C_21_H_19_O_1_^+^), was identified as apigenin-*O*-glucoronide based on its aglycone ion at *m/z* 271. Flavonol glycosides such as kaempferol-3-*O*-β-D-glucoside (S4 Fig) were reported to possess hepatoprotective action against damage induced by CCl_4_ *via* inhibiting lipid peroxidation caused by CCl_4_ reactive free radicals [60].

Three free flavonoid aglycones were detected in peaks 19, 21 and 24, corresponding to luteolin (*m/z* 285.0407, C_15_H_9_O_6_), kaempferol(*m/z* 287.0548, C_15_H_11_O_6_^+^) and apigenin(peak 24, *m/z* 269.0458, C_15_H_9_O_5_^-^) identified based on their retro-Diels-Alder (RDA) fragments through heterocyclic ring producing daughter ions at *m/z* 285, 287 and 269, respectively [61]. In addition, hydroxy-tetramethoxyflavone (peak 2, *m/z* 357.0869, C_19_H_17_O_7_^-^) was identified based on a fragment ion at *m/z* 299 [M-H-2CH_3_-CO]^-^ [62] showing losses of methyl groups.

#### 3.2.4. Terpenes.

Two terpenes were identified, i.e., sesquiterpene lactone; melampolide (costunolide) (peak 17; *m/z* 233.1500, C_15_H_21_O_2_^+^) based on fragment ions generated from the consecutive loss of H_2_O and CO leading to daughter ions at *m/z* 187 (C_14_H_19_^+^) and*m/z*159 (C_12_H_13_^+^) (S5 Fig), and a triterpene annotated as maslinic acid (peak 44; *m/z* 471.3479, C_30_H_47_O_4_^-^) based on its dehydrated fragment ion at *m/z* 455 as reported previously [63,64]. Maslinic acid was reported to prevent the generation of pro-inflammatory cytokines and oxidative stress *via* downregulating NF-κB and STAT-1 [64]. Melampolide (costunolide) is reported to inhibit the expression of NF-κB (nuclear factor kappa-light-chain-enhancer of activated B cells) by phosphorylation, which is directly related to its anti-inflammatory and anticancer activities [63].

#### 3.2.5. Fatty Acids.

Eight fatty acids encompassing polyunsaturated and hydroxylated forms represent one of the major classes identified in SOEtOH *i.e.*, octadecatetraenoic acid (peak 28; *m/z* 277.2158, C_18_H_29_O_2_^+^), hydroxynonadecadienoic acid (peak 20; *m/z* 311.2626, C_19_H_35_O_3_^+^), epoxy-hydroxy-octadecadienoic acid (peak 27; *m/z* 311.2186, C_18_H_31_O_4_^+^), hydroxy-oxo-phytodienoic acid (peak 25; *m/z* 307.1917, C_18_H_27_O_4_^-^) (S6 Fig), hydroxyoctadecadienoic acid (peak 30; *m/z* 295.2283, C_18_H_31_O_3_^-^), hydroxynonadecatrienoic acid (peak 31; *m/z* 309.2442, C_19_H_33_O_3_^+^), dihydroxyoctadecanoic acid (peak 26; *m/z* 287.2229, C_16_H_31_O_4_^-^) (S7 Fig) and trihydroxyoctadecadienoic acid (peak 18; *m/z* 327.218, C_18_H_31_O_5_). Hydroxylated fatty acids are well reported for their cytotoxic and anti-inflammatory activities [65] and whether they contribute to the observed effect of SOEtOH in mitigation against paracetamol hepatotoxic effect.

### 3.3. Molecular simulation results

#### 3.3.1. Molecular Operating Environment (MOE) results on JNK1 and JNK2 proteins.

The molecular docking studies of the main phenolic and flavonoids identified in the SOETOH were performed on c-Jun NH_2_-terminal kinases (s), justifying the observed hepatoprotective action exerted by the extract. The compounds showed remarkable binding affinities when compared to the co-crystallized reference for the investigated proteins (Table 2).

**Table 2:** Docking simulations results of the identified phenolics and flavonoids on JNK-1 and −2.

Compound	ΔG ^*a*^ (kcal/mol)	
**JNK1**	**JNK2**
Hydroxy-tetramethoxyflavone (**2**)	−7.14	−7.22
Protocatechuic acid-*O*- hexoside(**3**)	−6.68	−7.18
Caffeic acid-*O*-hexoside (**4**)	−6.87	−6.82
Dihydroxycoumarin-*O*-hexoside (**5**)	−6.47	−7.08
Caffeic acid (**6**)	−5.05	−5.22
Dihydroxycoumarin (**7**)	−4.87	−4.96
Luteolin-*O*-di-hexoside (**8**)	−8.55	−8.02
Kaempferol-*O*-hexoside (**9**)	−7.67	−8.39
Luteolin-*O*-hexoside (**10**)	−7.35	−7.68
3,4-Di-*O*-caffeoylquinic acid (**11**)	−7.86	−8.48
Isorhamnetin-*O*-hexoside (**12**)	−8.08	−7.91
Apigenin-*O*-glucoronide (**13**)	−7.23	−7.53
Apigenin (**17**)	−6.26	−6.45
Luteolin (**19**)	−6.33	−6.73
Kaempferol (**21**)	−5.81	−6.19
Caffeic acid ethyl ester (**22**)	−5.87	−6.10
The co-crystallized ligand	−6.67	−12.82

^a^ΔG: binding affinity

**Fig 7:**
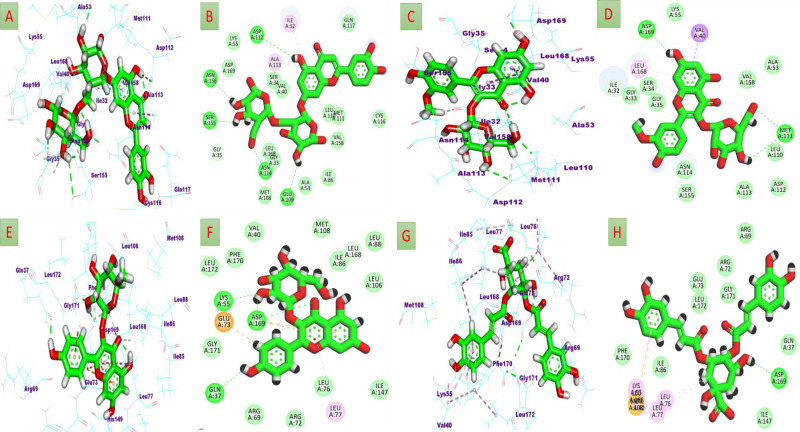
Binding mode in the active site of JNK1 (PDB ID: 3V3V) and JNK2 (PDB ID: 3NPC). (A, B) 3D and 2D binding mode of the luteolin-*O*-di-hexoside in the active site of JNK1, (C, D) 3D and 2D binding mode of the isorhamnetin-*O*-hexoside in the active site of JNK1, (E, F) 3D and 2D binding mode of the kaempferol-*O*-hexoside in the active site of JNK2, (G, H) 3D and 2D binding mode of the 3,4-di-*O*-caffeoylquinic acid in the active site of JNK2.

By comparing to the co-crystallized ligands for the examined proteins, the compounds displayed remarkable binding affinities. Regarding JNK1, luteolin-*O*-di-hexoside, and isorhamnetin-*O*-hexoside displayed the best binding affinity with ΔG = −8.55 and −8.08 kcal/mol, respectively. Several hydrogen bonds were formed between the hydroxyl groups in flavonoid aglycone and glycosidic moieties of luteolin-*O*-di-hexoside with the crucial amino acids Glu 109, Asn 114, Asn 156, Ser 155, and Asp 112. Also, π-alkyl interactions were presented between the C-ring of luteolin with Ala 113 (Fig 7A & B). Also, the hydroxyls of hexoside moiety in isorhamnetin-*O*-hexoside formed hydrogen bonds with Met 111. As well, B and C-rings of aglycone formed π-alkyl interactions with Asp 168 (Fig 7C & D), suggesting that both aglycon and sugar moieties contribute to binding. It is noteworthy that the previous studies revealed the ability of the flavone quercetagetin to inhibit JNK1 activity more potently than did the pharmacological inhibitor (SP600125) with IC_50_ values 4.6 μM and 5.2 μM, respectively. Additionally, as shown in animal models, quercetagetin inhibited UVB-induced phosphorylation of c-Jun and AKT in JB6 P_+_ cells [66]. These findings support the well affinity of flavone-based glycosides to JNK1.

Similarly, toward JNK2, the 3,4-di-*O*-caffeoylquinic and kaempferol-*O*-hexoside revealed the highest binding affinity with ΔG = −8.48 and −8.39 kcal/mol, respectively (Table 2). The glycosidic and phenolic hydroxyls of the latter compound formed four hydrogen bonds with Lys 55, Asp 169, and Gln 37 of JNK2 (Fig 7E & F). the other compound, 3,4-di-*O*-caffeoylquinic, formed hydrogen bonds with Asp 169 as well as π-alkyl and π-S interactions with other critical amino acids(Fig 7G & H).These results cope with the previous studies that reported the efficiency of the quercetin to suppress JNK phosphorylation (Perez-Vizcaino et al. 2006). As well as, quercetin-3-*O*-*β*-D glucuronide potently inhibited JNK activation [67].These findings support the good affinity of the flavonoid identified in the extract of *S. oleraceus* to JNK1 and 2.

#### 3.3.2. Molecular dynamic and system stability.

**Fig 8:**
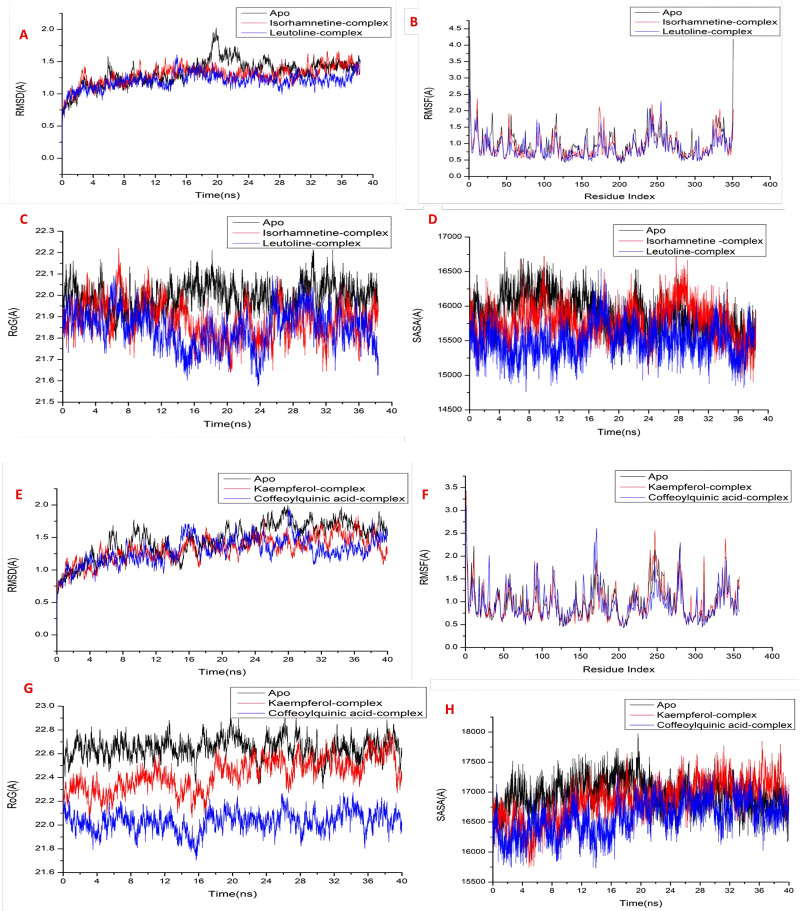
Results of Molecular Dynamic (MD) simulation and system stability on JNK1 and JNK2 protein. [**A**] RMSD of C*α* atoms of the protein backbone atoms on JNK1, [**B**] RMSF of each residue of the protein backbone Cα atoms of protein residues on JNK1, (**C**) ROG of Cα atoms of protein residues on JNK1, and (**D**) solvent accessible surface area (SASA) of the C*α* of the backbone atoms relative (black) to the starting minimized over 40 ns for the catalytic binding site with isorhamnetin-*O*-hexoside – JNK1 (red), and luteolin-*O*-di-hexoside-JNK1(blue), [**E**] RMSD of Cα atoms of the protein backbone atoms on JNK2, [**F**] RMSF of each residue of the protein backbone Cα atoms of protein residues on JNK2, (**G**) ROG of Cα atoms of protein residues on JNK2, and (**H**) solvent accessible surface area (SASA) of the Cα of the backbone atoms relative (black) to the starting minimized over 40 ns for the catalytic binding site with kaempferol-*O*-hexoside–JNK2 (red) and 3,4-di-*O*-caffeoylquinic acid–JNK2 (blue).

To forecast how the compounds would behave upon binding to the protein active site, as well as how they would interact and remain stable, a molecular dynamic simulation was run. To track disturbed motions and prevent artifacts from forming throughout the simulation, system stability was verified. The stability of the systems during the simulations was evaluated in this study using Root Mean Square Deviation (RMSD) [68]. For all frames of the apo-protein, isorhamnetin-*O*-hexoside-JNK1 and luteolin-O-di-hexoside-JNK1 systems, the recorded average RMSD values were 1.331 ± 0.19 Å, 1.29 ± 0.14 Å, and 1.20 ± 0.12 Å (Fig 8A-D). For the apo-protein, kaempferol-O-hexoside-JNK2 and 3,4-di-O-caffeoylquinic acid-JNK2 systems, the corresponding RMSD values were 1.46 ± 0.25 Å, 1.33 ± 0.19 Å, and 1.30 ± 0.20 Å (Fig 8E-HA). According to these findings, the protein complex system including luteolin-*O*-di-hexoside and 3,4-di-*O*-caffeoylquinic acid developed a comparatively more stable shape than the other systems under investigation.

Throughout MD modeling, evaluating the flexibility of protein structures upon ligand binding is essential for investigating residue behavior and their relationship to the ligand [69]. The Root-Mean-Square Fluctuation (RMSF) technique was used to assess protein residue changes in order to assess the impact of inhibitor binding to the corresponding targets during the simulations. The average RMSF values for all frames of the apo-protein, isorhamnetin-*O*-hexoside-JNK1 and luteolin-*O*-di-hexoside-JNK1 systems were 0.99 ± 0.43 Å, 0.91 ± 0.36 Å, and 0.86 ± 0.33 Å, respectively, as shown in Fig 8A-D. For the apo-protein, kaempferol-*O*-hexoside-JNK2 and 3,4-di-*O*-caffeoylquinic acid-JNK2 systems were 1.00 ± 0.39 Å, 0.97 ± 0.39 Å, and 0.96 ± 0.37 Å, respectively (Fig 8E-H). According to these results, the protein complex system containing luteolin-*O*-di-hexoside and 3,4-di-*O*-caffeoylquinic acid has less fluctuation than the other systems.

ROG was found to assess both the stability of the system following ligand binding and its overall compactness during MD simulation [70]. The average Rg values for all frames of the systems for the apo-protein, isorhamnetin-*O*-hexoside-JNK1 and luteolin-O-di-hexoside-JNK1 systems were 21.99 ± 0.06 Å, 21.88 ± 0.08 Å, and 21.84 ± 0.08 Å (Fig 8C). For the apo-protein, kaempferol-*O*-hexoside-JNK2 and 3,4-di-*O*-caffeoylquinic acid-JNK2 systems, the corresponding Rg values were 22.64 ± 0.08 Å, 22.43 ± 0.13 Å, and 22.03 ± 0.08 Å (Fig 8G). The behavior indicates that the complex of luteolin-*O*-di-hexoside and 3,4-di-*O*-caffeoylquinic acid is extremely rigid against the target receptor’s catalytic binding site.

The protein’s SASA was calculated in order to assess the compactness of the hydrophobic core. This was accomplished by measuring the protein’s solvent-visible surface area, which is crucial for the stability of biomolecules. Apo-protein, isorhamnetin-*O*-hexoside-JNK1, and luteolin-O-di-hexoside-JNK1 systems had average SASA values of 15892.56 Å, 15771.56 Å, and 15500.04 Å, respectively (Fig 8D). Apo-protein, kaempferol-*O*-hexoside-JNK2, and 3,4-di-*O*-caffeoylquinic acid-JNK2 systems had average SASA values of 16948.83 Å, 16891.38 Å, and 16583.11 Å (Fig 8H). The luteolin-*O*-di-hexoside and 3,4-di-*O*-caffeoylquinic acid complex system continue intact inside the catalytic binding region of target receptors, as confirmed by the SASA result when combined with the findings from the RMSD, RMSF, and ROG calculations.

#### 3.3.3. Binding interaction mechanism based on binding free energy calculation.

Combining the generalized Born and surface area continuum solvation, the molecular mechanics energy methodology (MM/GBSA) is a widely used approach for calculating the free binding energies of small molecules to biological macromolecules and may be more reliable than docking scores. The binding free energies were determined by taking snapshots of the systems’ trajectories and using the MM-GBSA function in AMBER18. All of the reported calculated energy components (except from ΔG_solv_) provided high negative values, indicating positive interactions, as Table 3 illustrates.

**Table 3:** Calculated energy binding for the extracted compound against the catalytic binding site of JNK1 and JNK2.

Energy Components (kcal/mol)					
JNK1					
Complex	ΔE_vdW_	ΔE_elec_	ΔG_gas_	ΔG_solv_	ΔG_bind_
Isorhamnetin-*O*-hexoside	−42.92 ± 0.49	−33.57 ± 0.77	−76.50 ± 0.63	31.30 ± 0.43	−45.19 ± 0.46
Luteolin-*O*-di-hexoside	−48.61 ± 0.59	−54.13 ± 1.31	−102.74 ± 1.23	54.47 ± 0.77	−48.27 ± 0.72
**JNK2**					
Complex	**ΔE**_**vdW**_	**ΔE**_**elec**_	**ΔG**_**gas**_	**ΔG**_**solv**_	**ΔG**_**bind**_
Kaempferol-*O*-hexoside	−63.78 ± 0.49	−17.43 ± 1.32	−81.12 ± 1.33	34.99 ± 1.29	−46.12 ± 0.61
3,4-di-*O*-Caffeoylquinic acid	−47.39 ± 0.66	−67.04 ± 1.21	−114.44 ± 1.27	59.62 ± 0.74	−54.81 ± 0.79

∆E_vdW_ = van der Waals energy; ∆E_elec_ = electrostatic energy; ∆G_solv_ = solvation free energy; ∆G_bind_ = calculated total binding free energy.

According to a thorough analysis of each individual energy contribution, the more positive Vander Waals energy component drives the interactions between isorhamnetin-*O*-hexoside, kaempferol-*O*-hexoside, and the target protein receptor residues, while the more positive Electrostatic energy component drives the interactions between luteolin-*O*-di-hexoside, 3,4-di-*O*-caffeoylquinic acid, and the target protein receptor residues. These interactions are demonstrated by the binding free energies found in Table 3.

#### 3.3.4. Identification of the critical residues responsible for ligands binding.

**Fig 9:**
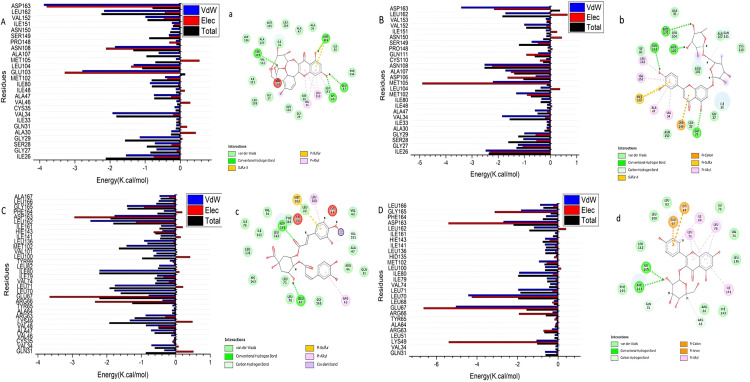
Per-residue decomposition plots. Showing the energy contributions to the binding and stabilization of isorhamnetin-*O*-hexoside [**A**], and luteolin-*O*-di-hexoside [**B**] into catalytic binding site of JNK-1, as well as kaempferol-*O*-hexoside [**C**], and 3,4-di-*O*-caffeoylquinic acid [**D**] into catalytic binding site of JNK-2. Corresponding inter-molecular interactions are shown [a], [b], [c], and [d].

To have a better understanding of the key residues involved in the suppression of the catalytic binding site receptor, the total energy involved when a drug binds these enzymes was further broken down into the involvement of particular site residues. Based on (Fig 9A), the significant beneficial contributions of isorhamnetin-*O*-hexoside to the catalytic binding site of JNK1 receptor were predominantly observed from residues Ile26 (−1.54 kcal/mol), Gly27 (−0.674 kcal/mol), Ser28 (−0.718 kcal/mol), Gly29 (−1.149 kcal/mol), Ala30 (−0.25 kcal/mol), Gln31 (−0.11 kcal/mol), Val34 (−1.924 kcal/mol), Ala47 (−0.744 kcal/mol), Ile48 (−0.124 kcal/mol), Ile80 (−0.859 kcal/mol), Met102 (−0.429 kcal/mol), Glu103 (−2.783 kcal/mol), Leu104 (−1.323 kcal/mol), Ala107 (−0.611 kcal/mol), Asn108 (−1.825 kcal/mol), Ser149 (−0.319 kcal/mol), Asn150 (−0.243 kcal/mol), Ile151 (−0.234 kcal/mol), Val152 (−0.988 kcal/mol), Leu162 (−2.164 kcal/mol), and Asp163 (−3.879 kcal/mol).

Otherwise, the significant favorable contributions of luteolin-*O*-di-hexoside to the catalytic binding site of JNK1 were primarily noticed from residues Ile26 (−2.474 kcal/mol), Gly27 (−1.242 kcal/mol), Ser28 (−1.037 kcal/mol), Gly 29 (−0.994 kcal/mol), Ala30 (−0.145 kcal/mol), Ile33 (−0.138 kcal/mol), Val34 (−1.843 kcal/mol), Ala47 (−0.591 kcal/mol), Ile48 (−0.122 kcal/mol), Ile80 (−0.341 kcal/mol), Met102 (−1.306 kcal/mol), Leu104 (−0.373 kcal/mol), Met105 (−2.196 kcal/mol), Asp106 (−0.559 kcal/mol), Ala107 (−1.721 kcal/mol), Asn108 (−2.514 kcal/mol), Gln111 (−0.466 kcal/mol), Ser149 (−0.714 kcal/mol), Asn150 (−0.157 kcal/mol), Ile151 (−0.172 kcal/mol), Val152 (−0.996 kcal/mol), Leu162 (−1.72 kcal/mol), and Asp 163 (−3.408 kcal/mol) (Fig 9B).

Moreover, from Fig 9C, the major good contributions of kaempferol-*O*-hexoside with the catalytic binding site of JNK2 were mainly witnessed from residues Gln31 (−0.636 kcal/mol), Val34 (−0.116 kcal/mol), Lys49 (−1.058 kcal/mol), Arg63 (−0.297 kcal/mol), Arg 66 (−0.266 kcal/mol), Ile 33 (−0.138 kcal/mol), Val 34 (−1.843 kcal/mol), Ala 47 (−0.591 kcal/mol), Glu67 (−5.033 kcal/mol), Leu 70 (−4.423 kcal/mol), Leu71 (−1.96 kcal/mol), Val74 (−0.382 kcal/mol), Ile79 (- 0.545 kcal/mol), Ile 80 (−1.911 kcal/mol), Leu 100 (−0.633 kcal/mol), Met102 (−0.937 kcal/mol), Leu136 (−0.634 kcal/mol), Ile141 (−0.646 kcal/mol), Hie143 (−0.635 kcal/mol), Ile161 (−0.146 kcal/mol), Leu162 (−1.183 kcal/mol), Asp163 (−2.688 kcal/mol), Gly165 (−1.817 kcal/mol), and Leu166 (−0.881 kcal/mol).

Ultimately, the essential favorable contributions of 3,4-di-*O*-caffeoylquinic acid towards the catalytic binding site of JNK2 were essentially found from residues Gln31 (−0.331 kcal/mol), Val34 (−0.704 kcal/mol), Ala47 (−0.715 kcal/mol), Val48 (−0.863 kcal/mol, Lys49 (−1.332 kcal/mol), Arg63 (−0.872 kcal/mol), Arg 66 (−0.384 kcal/mol), Ile 33 (−0.138 kcal/mol), Val 34 (−1.843 kcal/mol), Ala 47 (−0.591 kcal/mol), Glu67 (−0.785 kcal/mol), Leu70 (−1.78 kcal/mol), Leu71 (−1.76 kcal/mol), Val74 (−0.549 kcal/mol), Ile79 (- 0.538 kcal/mol), Ile80 (−2.212 kcal/mol), Leu82 (−0.479 kcal/mol), Leu100 (−0.641 kcal/mol), Val101 (−0.658 kcal/mol), Met102 (−1.426 kcal/mol), Le 136 (−0.91 kcal/mol), Ile141 (−0.433 kcal/mol), Hie 143 (−0.338 kcal/mol), Ile161 (−0.423 kcal/mol), Leu162 (−2.471 kcal/mol),), Asp163 (−1.775 kcal/mol), Gly165 (−1.414 kcal/mol), Leu166 (−0.491 kcal/mol), Ala167 (−0.466 kcal/mol) (Fig 9D).

## 4. Discussion

It is proven that the liver is the major organ that is subjected to drug toxicity due to many concerns [71]. the intracellular enzymes such as (ALT and AST) are found inside the liver, heart, and muscles and are typically used to assess for liver cellular damage [72]. Elevated levels of ALT and AST are frequently observed following the administration of high doses of paracetamol, which is a leading cause of such increases [73]. The extent of this elevation can offer valuable information regarding the severity of liver injury, with higher levels typically indicating more significant liver damage. The role of flavonoids the primary constituents of SOEtOH, has been well established in ameloration of the aminotransferases owing to their scavenging activities [74].

Mitochondria play the major target for xenobiotic-induced bioenergetic failure. making them the most liable organelle to oxidative stress [75]. Superoxide is generated within the mitochondrial matrix due to electron leakage from the electron transport chain, a process initiated by protein adduct formation and then enhanced by the mitochondrial translocation of phospho-JNK. These superoxide radicals can combine to nitric oxide forming peroxynitrite, a potent oxidant and nitrating agent [5]. Peroxynitrite contributes significantly to oxidative stress by nitrating proteins and plays a critical role in paracetamol-induced cellular toxicity [76]. Glutathione (GSH) is responsible for scavenging peroxynitrite, and its depletion is a key indicator of acetaminophen toxicity, driven by the reactive metabolites of paracetamol [77]. Protein nitration inactivates mitochondrial-specific superoxide dismutase 2 (MnSOD), which normally converts superoxide to hydrogen peroxide and oxygen, thereby exacerbating oxidative damage [78]. MnSOD has been shown to provide strong protection against paracetamol-induced liver injury in mice, even when administered post-metabolism, due to its similarity to human paracetamol pathophysiology [79]. As mentioned above, SOD has been supposed as a potential and therapeutic target for mitigating liver damage.

Also, it is noted that the Kelch-like ECH-associated protein 1 (Keap1)/erythroid 2-related factor 2 (Nrf2)/heme oxygenase-1 (HO-1) is a crucial pathway axis in defense against paracetamol-induced liver injury [80]. Nrf2 plays its role in the antioxidant process by regulating the antioxidant genes and drug-metabolizing enzymes, as well as attaching to the antioxidant response elements (AREs) in their promoters, lowering the hepatotoxic effects of paracetamol [81]. The role of Keap1 came to be the main negative regulator of Nrf2. So, when Nrf2 is activated, Keap1 is released, which causes the production of several genes involved in antioxidant and detoxification [82]. The role of HO-1 in this pathway axis is found to facilitate heme lysis, speeding up the generation of biliverdin and lowering intracellular ROS levels. Paracetamol’s liver damage is mostly brought on by oxidative stress. The Keap1/Nrf2/HO-1 axis may aid in protecting against paracetamol-induced liver damage since Nrf2 is crucial in the defense against oxidative stress [83].

Based on the available evidence, the various Sonchus plant extracts shown notable hepatoprotective effects against a number of liver-toxicities, including paracetamole. Strong harmonical relationships were found between the current results with earlier evidence on the hepatoprotective properties of Sonchus plants, including *Sonchus arvesis* [84], *Sonchus cornutus* [46], and *Sonchus asper* [85].

Flavonoids have been shown in multiple studies to play an advisory role in modifying NRF2 through a variety of methods, which lowers ROS levels in the liver [86]. Luteolin reduces liver damage to varied degrees by blocking the TXNIP-NLRP3 axis, influencing HMGB1 release, encouraging the Nrf2/ARE pathway, and triggering the NF-κB cell apoptosis signaling pathway. By influencing the AMPK pathway and enhancing SIRT1 activity, luteolin also lessens alcohol-induced steatosis and inflammation and slows the progression of alcoholic liver disease [87]. Apigenin can enhance hepatic health in cases of severe liver illness by upregulating the BCL-2 apoptotic pathway and downregulating Nrf2-signaling. Caffeic acid is useful in treating severe liver problems and has demonstrated possible antioxidant and anti-inflammatory qualities. By interfering with the Nrf2 binding site and preventing it from binding to Keap1, it can alter the expression of kelch-like ECH-associated protein-1 (Keap1), a liver cancer factor, and increase the expression of essential antioxidative signals like HO-1 [88]. According to Yao research, terpenoids help treat NAFLD primarily by controlling inflammation, oxidative stress, insulin resistance, and lipid metabolism disorders. The primary targets for terpenoid therapy are the AMPK, PPARs, Nrf-2, and SIRT 1 pathways [87].

**Fig 10:**
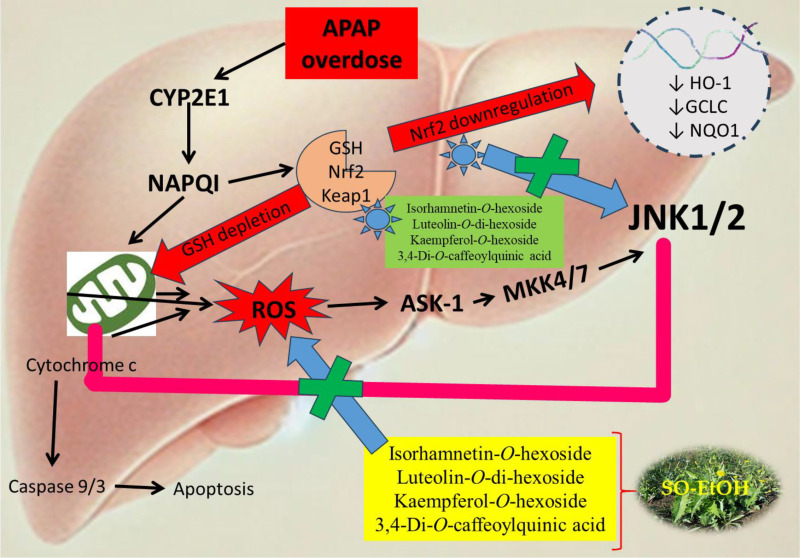
The mechanistic pathway associated with JNK1 and JNK2 proteins.

The JNK pathway is a pivotal regulator of cellular responses to stress, inflammation, and proliferation. Its modulation holds therapeutic potential across various diseases, particularly those characterized by inflammation and neurodegeneration. Active flavonoids such as luteolin and luteolin-O-di-hexoside have been shown to inhibit JNK activation, reducing oxidative stress and inflammatory responses, thus preventing pro-apoptotic signaling. [89,90]. This makes them promising candidates for treating neurodegenerative diseases and inflammatory disorders. Specifically, luteolin derivatives suppress JNK phosphorylation, hindering the activation of transcription factors like c-Jun and ATF-2, which are crucial in inflammation and apoptosis. Studies suggest that these compounds protect neurons in models of Parkinson’s and Alzheimer’s diseases by modulating JNK signaling [91]. Similarly, isorhamnetin and its glycosylated form, isorhamnetin-O-hexoside, have demonstrated anti-inflammatory and anticancer effects. Isorhamnetin inhibits JNK activation, reducing inflammation and apoptosis by targeting upstream kinases like ASK1 and MKK4/7. Its glycosylated derivative may enhance solubility and cellular uptake, further improving its therapeutic potential. Isorhamnetin’s ability to modulate JNK signaling underscores its role in treating cardiovascular diseases and cancer (Fig 10) [92,93].Another notable compound, 3,4-di-O-caffeoylquinic acid, exhibits strong anti-inflammatory and antioxidant properties. It mitigates oxidative stress-induced JNK activation by scavenging reactive oxygen species (ROS), effectively reducing inflammatory and apoptotic signaling. [94]. This compound has shown promise in treating chronic inflammatory disorders and offers neuroprotective benefits in cerebral ischemia models [95]. Kaempferol, along with its glycosylated forms, notably kaempferol-O-hexoside, also inhibits JNK signaling, especially in cancer cells. This inhibition suppresses cell proliferation and enhances apoptosis, potentially limiting tumor growth and inflammation. [96]. Kaempferol’s action on JNK signaling has been highlighted in cancer models, including colorectal and breast cancers, where it contributes to tumor suppression (Fig 10) [97]. Collectively, these compounds modulate the JNK pathway via different mechanisms, reducing inflammation, oxidative stress, and apoptosis, positioning them as promising therapeutic agents for diseases driven by JNK dysregulation.

The study on the phenolic and flavonoid compounds in *S. oleraceus* highlights their strong binding affinity to JNK1 and JNK2, demonstrating their potential as hepatoprotective agents. Molecular docking and dynamics simulations revealed that compounds like luteolin-*O*-di-hexoside and isorhamnetin-*O*-hexoside bind efficiently to JNK1, while 3,4-di-*O*-caffeoylquinic acid and kaempferol-*O*-hexoside show strong interactions with JNK2. These compounds, through various interactions such as hydrogen bonds and π-alkyl forces, show promising results compared to known inhibitors. However, clinical translation faces significant challenges, especially regarding pharmacokinetics and bioavailability. The complex structures and glycosidic nature of these flavonoids may hinder their absorption and stability, requiring further optimization. Despite the promising *in silico* results, experimental validation in preclinical models is crucial for confirming their therapeutic efficacy and safety, making pharmacokinetic considerations essential for developing effective JNK inhibitors

Current study bears two notable limitations. First, the absence of N-acetylcysteine (NAC) as a reference standard limited the comparative assessment of SOEtOH’s efficacy, despite NAC’s established role in oxidative stress models. Second, reliance on ELISA alone to assess Nrf2, KEAP-1, and HO-1 expression—without RT-PCR confirmation at the mRNA level—narrowed the molecular insight. Future research should address both gaps to bolster analytical rigor. Future research could explore synergistic studies combining *S. oleraceus* with standard hepatoprotective drugs to enhance therapeutic efficacy, optimize liver protection, and uncover potential mechanisms of action through collaborative pharmacological and molecular investigations.

## 5. Conclusion

This study provides the first evidence of the hepatoprotective potential of *S. oleraceus* ethanol extract (SOEtOH) against paracetamol-induced liver toxicity. Our findings demonstrate that SOEtOH significantly reduces elevated liver enzymes (ALT and AST), enhances antioxidant defense mechanisms (GSH and SOD), and mitigates histological damage in a dose-dependent manner. The extract also suppresses caspase-3 expression, suggesting its role in inhibiting apoptosis. On a molecular level, SOEtOH exerts its protective effects through the Nrf2/KEAP-1/HO-1 signaling pathway. Furthermore, UPLC-PDA-ESI-qTOF-MS/MS analysis revealed the presence of bioactive compounds, including flavonoids, phenolic compounds, fatty acids, and terpenes, which likely contribute to its hepatoprotective activity. Molecular docking studies highlighted that flavonoid and phenolic constituents exhibit strong binding affinities to JNK1 and JNK2, with luteolin-*O*-di-hexoside, isorhamnetin-*O*-hexoside, 3,4-di-*O*-caffeoylquinic acid, and kaempferol-*O*-hexoside showing the highest interactions. Overall, our results suggest that *S. oleraceus*, an edible plant, holds promise as a natural therapeutic agent for protecting against paracetamol-induced liver injury. Its bioactive compounds may offer a novel and effective strategy for liver protection, warranting further investigation for potential clinical applications.

## Supporting information

The following supporting information can be downloaded at: ………,

Fig S1 Tandem MS spectrum of peak 4, Rt. 8.092 min.
(DOCX)

Fig S2 Tandem MS spectrum of peak 11, Rt. 10.419 min.
(DOCX)

Fig S3 Tandem MS spectrum of peak 8, Rt. 9.603 min.
(DOCX)

Fig S4 Tandem MS spectrum of peak 9, Rt. 10.154 min.
(DOCX)

Fig S5 Tandem MS spectrum of peak 17, Rt. 11.404 min.
(DOCX)

Fig S6 Tandem MS spectrum of peak 25, Rt. 13.146 min.
(DOCX)

Fig S7 Tandem MS spectrum of peak 14, Rt. 10.802 min.
(DOCX)
