# Supplementary figures and images for: Hepatoprotective action of Sonchus oleraceus against paracetamol-induced toxicity via Nrf2/KEAP-1/HO-1 pathway in relation to its metabolite fingerprint and in silico studies
Source: PLoS One. 2025 Jun 26;20(6):e0325782. doi: 10.1371/journal.pone.0325782 (PMC12200742; doi:10.1371/journal.pone.0325782)

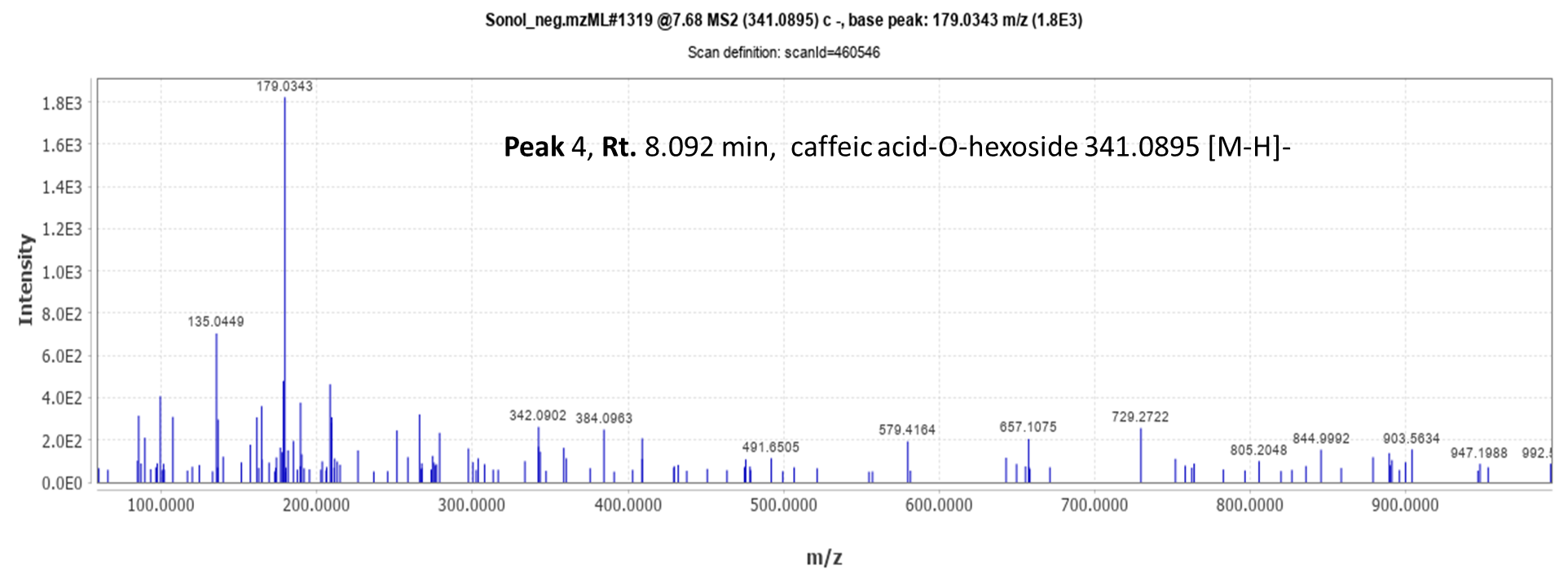


**Suppl. Fig. S1:** Tandem MS spectrum of peak 4, Rt. 8.092 min

Supplement: Fig S1 — 8.092 min. (DOCX) [file pone.0325782.s001.docx]

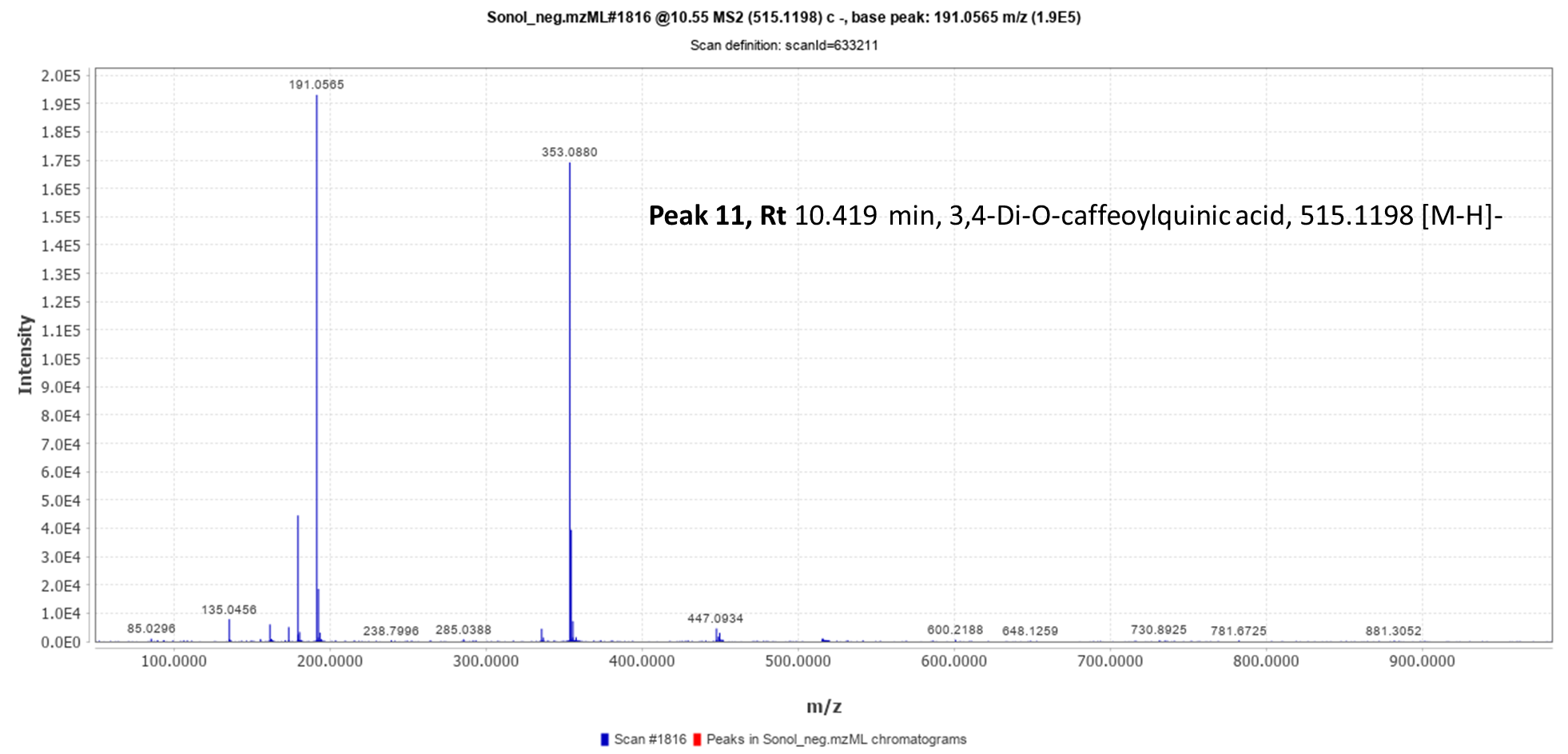


**Suppl. Fig. S2:** Tandem MS spectrum of peak 11, Rt. 10.419 min

Supplement: Fig S2 — 10.419 min. (DOCX) [file pone.0325782.s002.docx]

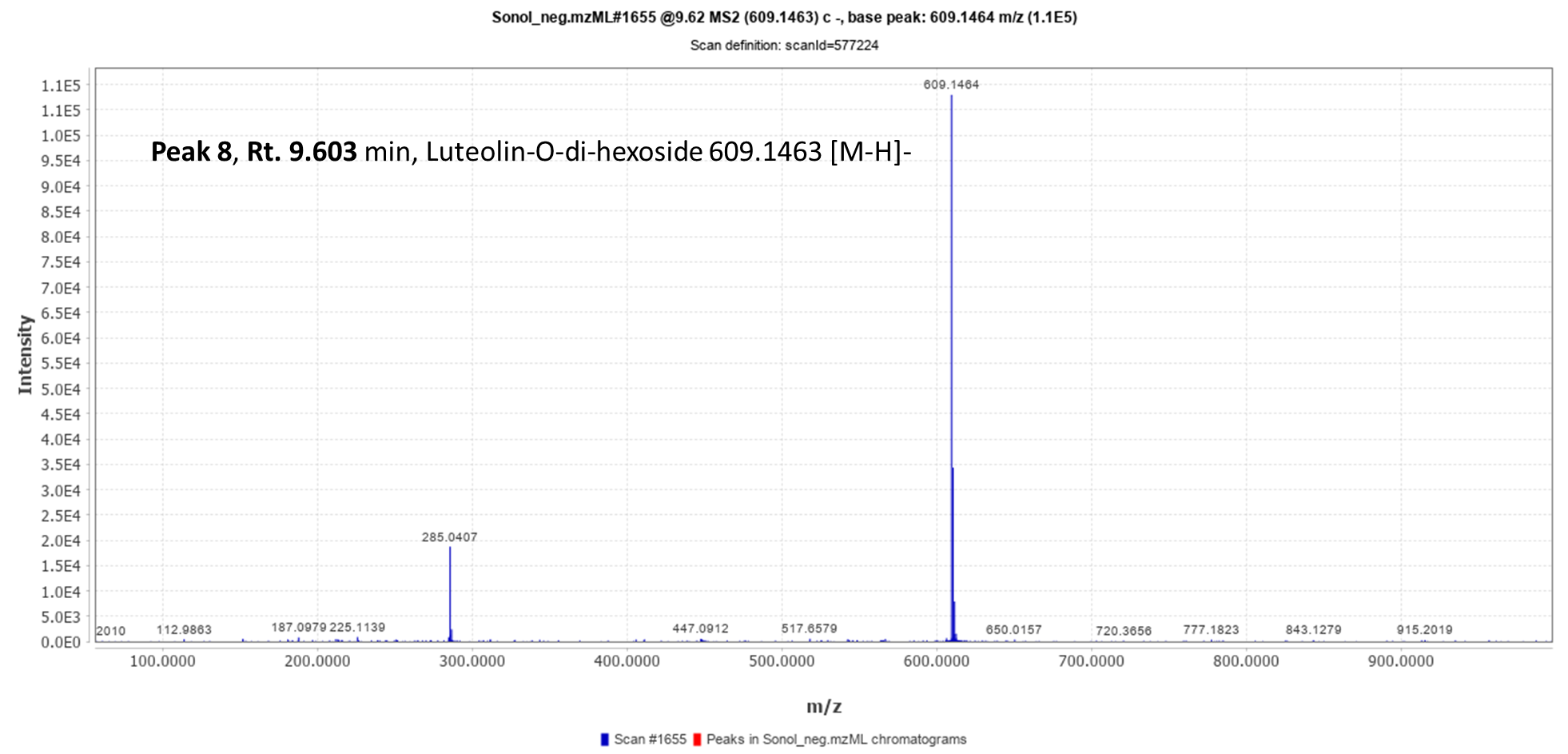


**uppl. Fig. S3:** Tandem MS spectrum of peak 8, Rt. 9.603 min

Supplement: Fig S3 — 9.603 min. (DOCX) [file pone.0325782.s003.docx]

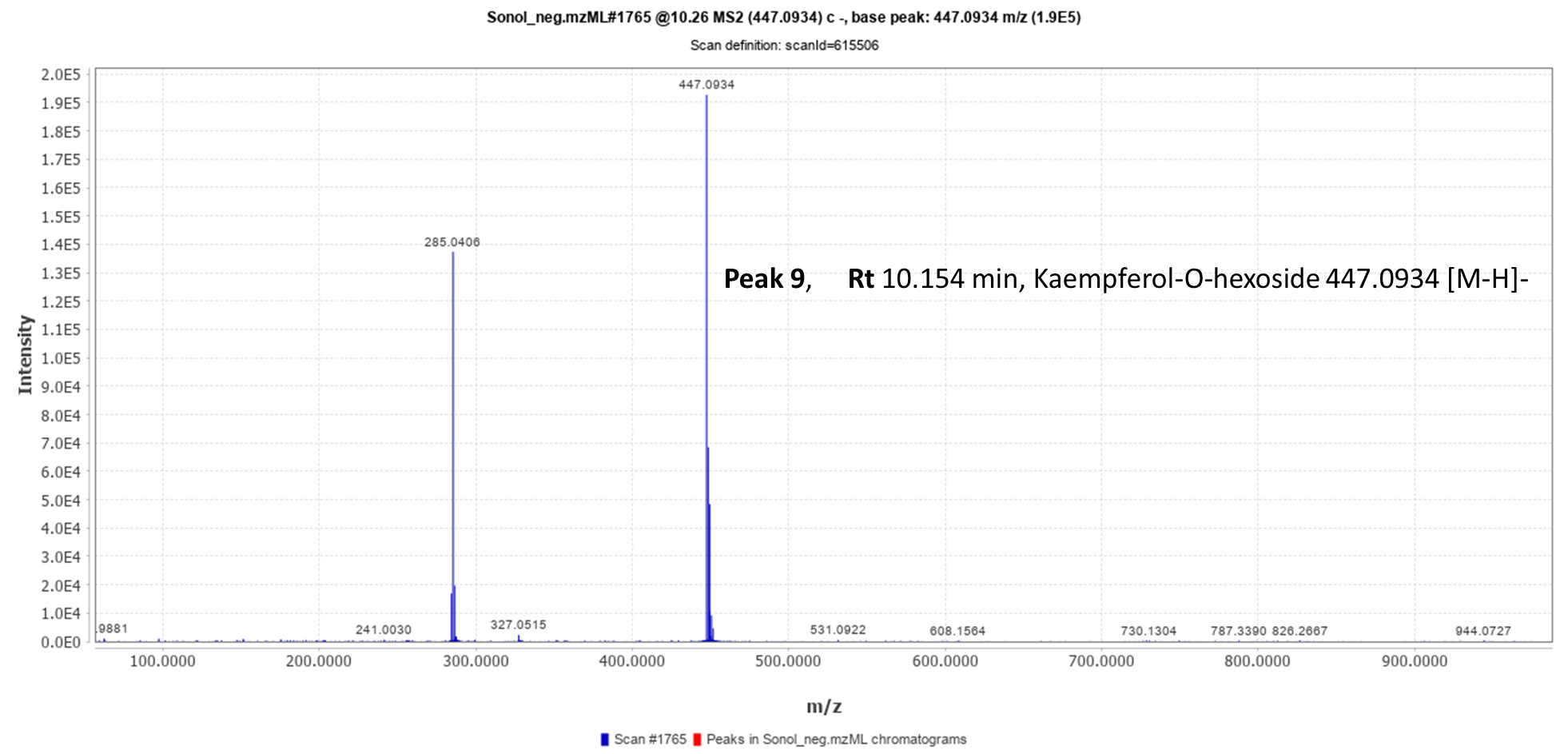


**Suppl. Fig. S4:** Tandem MS spectrum of peak 9, Rt. 10.154 min

Supplement: Fig S4 — 10.154 min. (DOCX) [file pone.0325782.s004.docx]

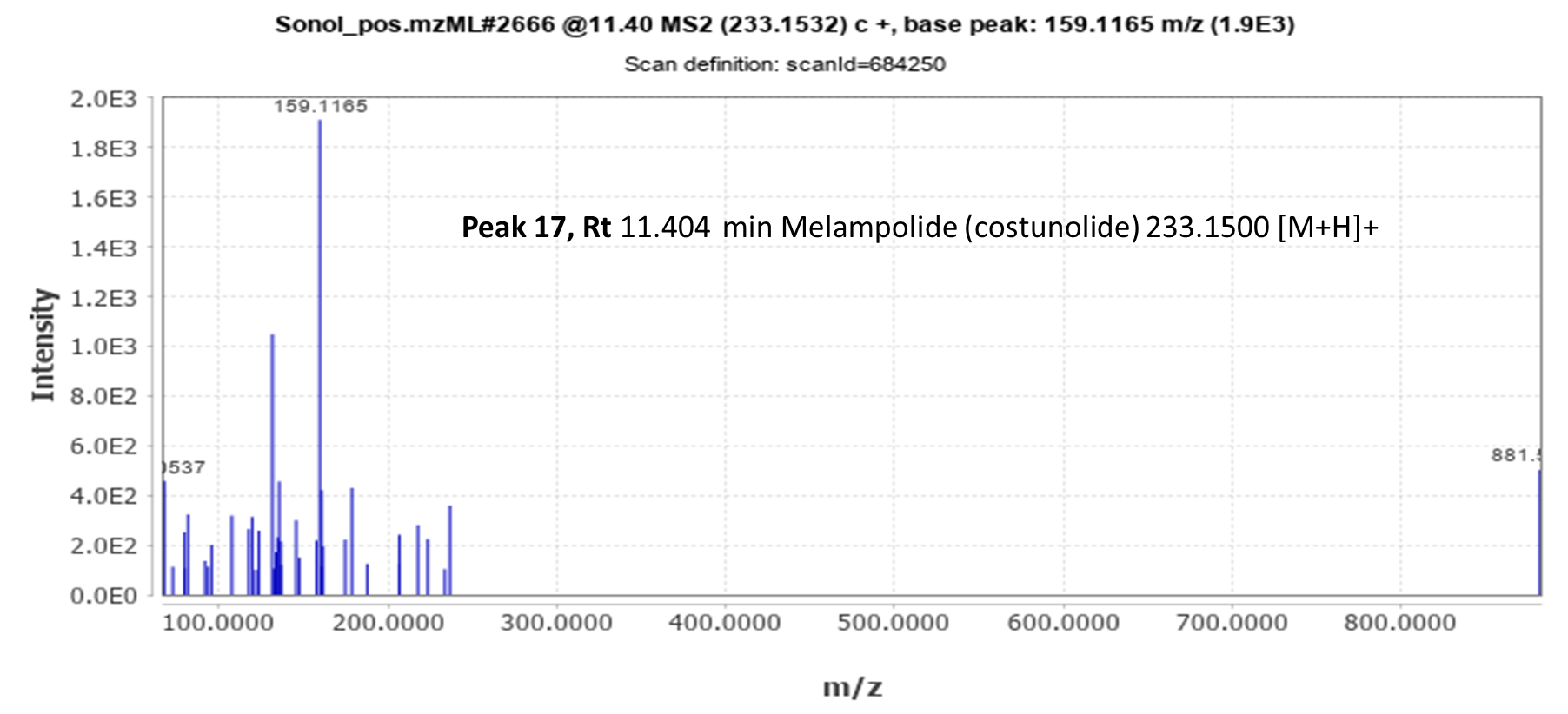


**Suppl. Fig. S5:** Tandem MS spectrum of peak 17, Rt. 11.404 min

Supplement: Fig S5 — 11.404 min. (DOCX) [file pone.0325782.s005.docx]

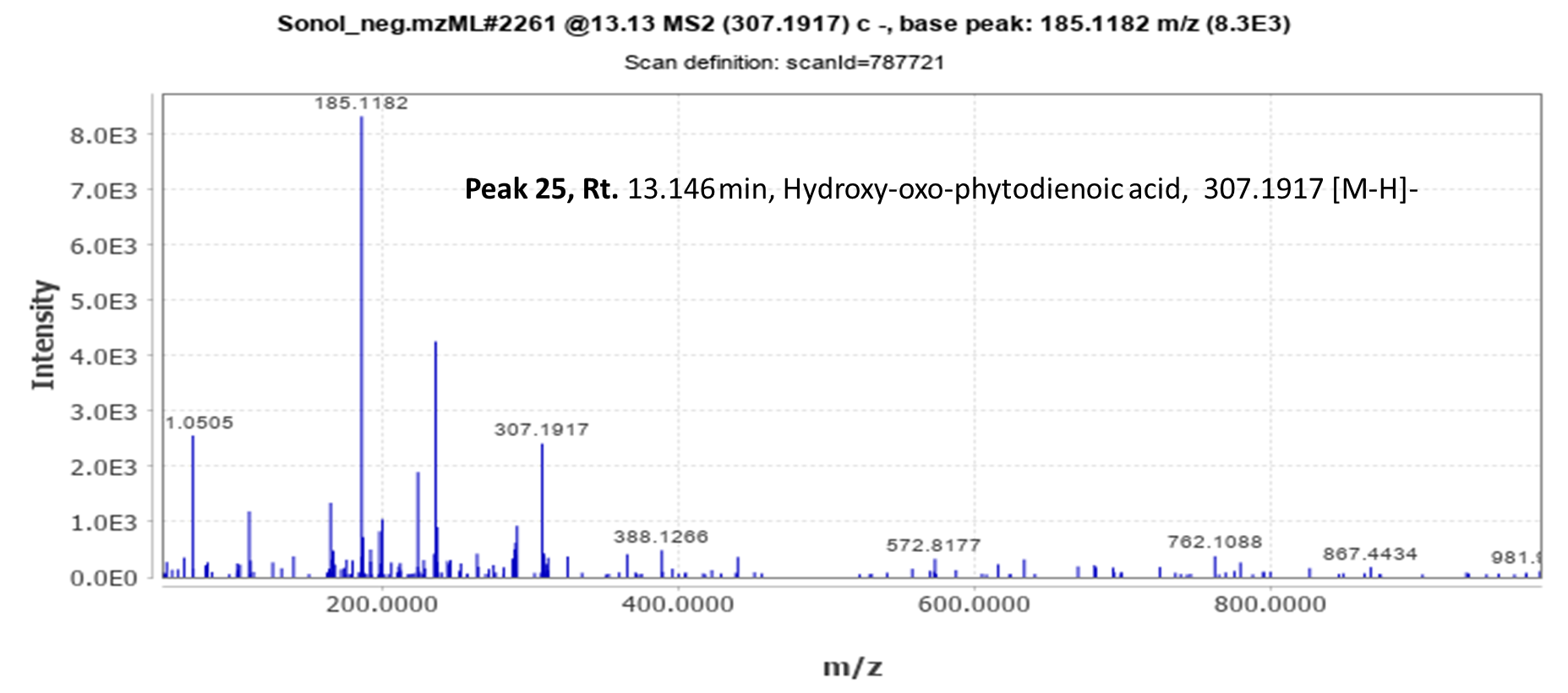


**Suppl. Fig. S6:** Tandem MS spectrum of peak 25, Rt. 13.146 min

Supplement: Fig S6 — 13.146 min. (DOCX) [file pone.0325782.s006.docx]

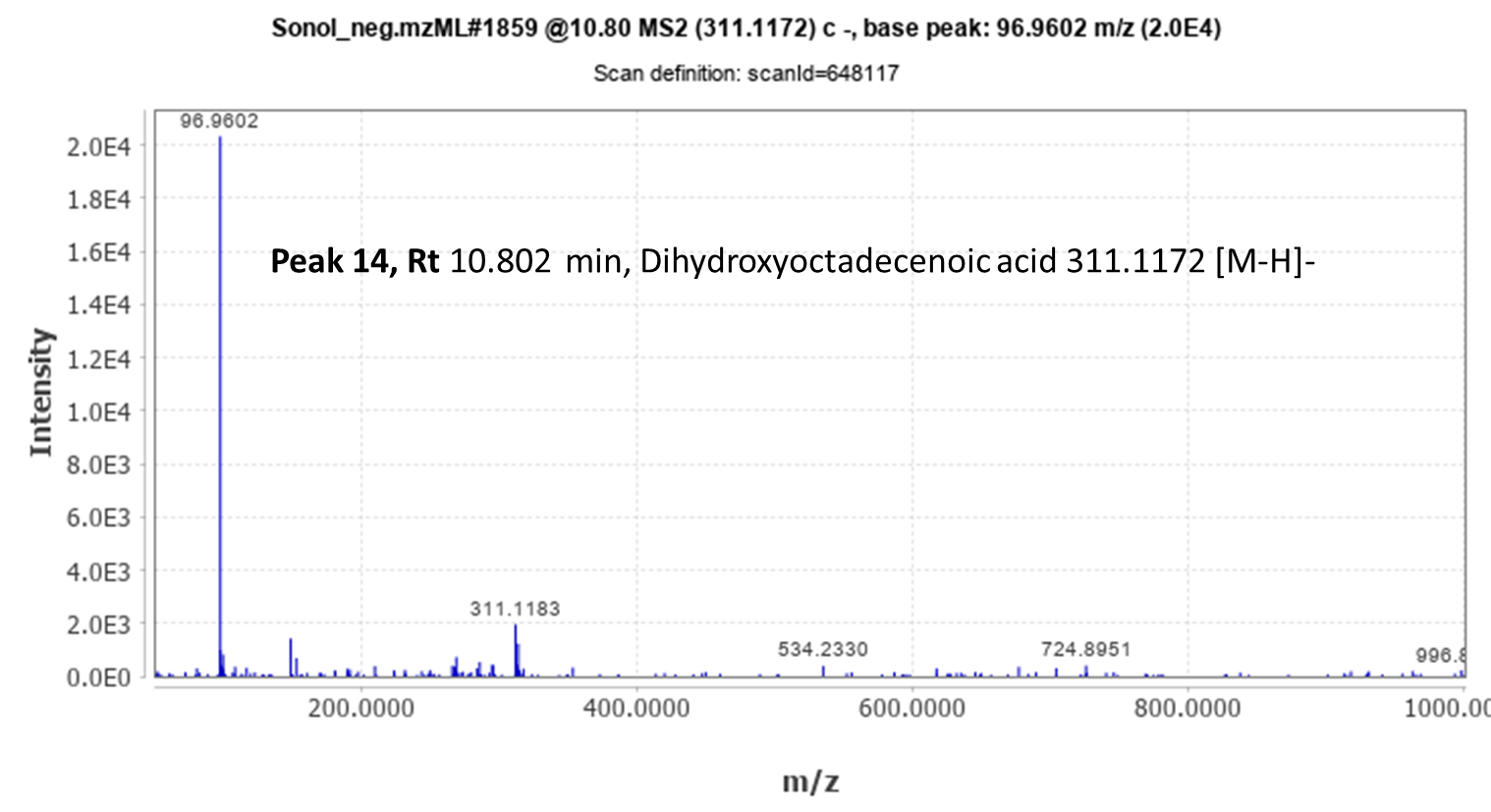


**Suppl. Fig. S7:** Tandem MS spectrum of peak 14, Rt. 10.802 min

Supplement: Fig S7 — 10.802 min. (DOCX) [file pone.0325782.s007.docx]
